# Convolutional Neural Network-Based Diagnostic Model for a Solid, Indeterminate Solitary Pulmonary Nodule or Mass on Computed Tomography

**DOI:** 10.3389/fonc.2021.792062

**Published:** 2021-12-21

**Authors:** Ke Sun, Shouyu Chen, Jiabi Zhao, Bin Wang, Yang Yang, Yin Wang, Chunyan Wu, Xiwen Sun

**Affiliations:** ^1^ Department of Radiology, Huashan Hospital, Fudan University, Shanghai, China; ^2^ Department of Radiology, Shanghai Pulmonary Hospital, Tongji University School of Medicine, Shanghai, China; ^3^ Department of Computer Science and Technology, College of Electronics and Information Engineering, Tongji University, Shanghai, China; ^4^ Department of Pathology, Shanghai Pulmonary Hospital, Tongji University School of Medicine, Shanghai, China

**Keywords:** neural network model, computed tomography, differential diagnosis, solid, indeterminate solitary pulmonary nodule, lung adenocarcinoma

## Abstract

**Purpose:**

To establish a non-invasive diagnostic model based on convolutional neural networks (CNNs) to distinguish benign from malignant lesions manifesting as a solid, indeterminate solitary pulmonary nodule (SPN) or mass (SPM) on computed tomography (CT).

**Method:**

A total of 459 patients with solid indeterminate SPNs/SPMs on CT were ultimately included in this retrospective study and assigned to the train (n=366), validation (n=46), and test (n=47) sets. Histopathologic analysis was available for each patient. An end-to-end CNN model was proposed to predict the natural history of solid indeterminate SPN/SPMs on CT. Receiver operating characteristic curves were plotted to evaluate the predictive performance of the proposed CNN model. The accuracy, sensitivity, and specificity of diagnoses by radiologists alone were compared with those of diagnoses by radiologists by using the CNN model to assess its clinical utility.

**Results:**

For the CNN model, the AUC was 91% (95% confidence interval [CI]: 0.83–0.99) in the test set. The diagnostic accuracy of radiologists with the CNN model was significantly higher than that without the model (89 *vs.* 66%, P<0.01; 87 *vs.* 61%, P<0.01; 85 *vs.* 66%, P=0.03, in the train, validation, and test sets, respectively). In addition, while there was a slight increase in sensitivity, the specificity improved significantly by an average of 42% (the corresponding improvements in the three sets ranged from 43, 33, and 42% to 82, 78, and 84%, respectively; P<0.01 for all).

**Conclusion:**

The CNN model could be a valuable tool in non-invasively differentiating benign from malignant lesions manifesting as solid, indeterminate SPNs/SPMs on CT.

## 1 Introduction

With the use of thoracic low-dose computed tomography (CT) for lung cancer screening, an increasing number of solitary pulmonary nodules (SPNs) or masses (SPMs) are deliberately or incidentally discovered. Solid SPNs are extremely common, and malignancy account for approximately 60% (range: 55–66%) (1, 2). Data from the Prostate, Lung, Colorectal, Ovarian Cancer Screening Trial indicated that SPMs were highly predictive of malignancy (odds ratio, 10.3; 95% confidence interval [CI], 2.46–43.38) (3). Solid malignant lesions are related to rapid cancer growth and high risks of recurrence and metastasis, despite their small size (4, 5). Therefore, the most crucial task for radiologists and clinicians is to accurately determine the natural history of the lesions. Surgery is the diagnostic gold standard and definitive treatment for malignant cases. However, 25–46% of patients with SPNs have benign disease despite a preoperative suspicion of cancer, and an incorrect diagnosis results in unnecessary invasive resection and monetary and time costs (6, 7).

**Figure 1:**
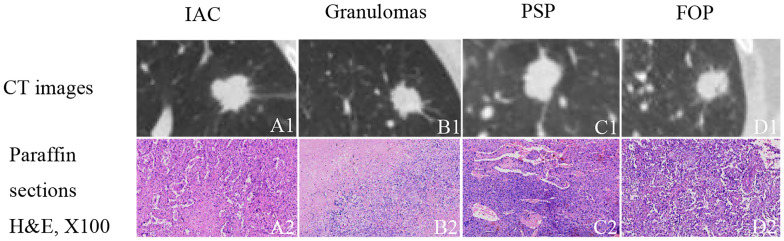
Examples of solid, indeterminate SPN/SPMs without features strongly suggestive of a benign etiology. **(A1)** Invasive adenocarcinoma (IAC); **(B1)** granuloma; **(C1)** pulmonary sclerosing pneumocytoma (PSP); **(D1)** focal organizing pneumonia (FOP). **(A2–D2)** paraffin section (hematoxylin and eosin [H&E], 100 ×) of IAC, granuloma, PSP, and FOP, respectively.

High-resolution computed tomography (HRCT) can non-invasively provide specific information about pulmonary lesions (8). However, there are challenges associated with the visual assessment of CT images. First, a series of CT images consist of hundreds of slices; radiologists have to browse through these slices and carefully consider them, which is time-consuming, tedious, and subjective. Second, visual evaluations are inadequate to distinguish benign from malignant lesions manifesting as solid, indeterminate SPNs or SPMs because of the considerable overlap in the radiographic characteristics of these lesion types (**Figure 1**). For example, 21–58% of malignant lesions have smooth edges, and approximately 25% of benign nodules are irregularly shaped with spiculated or lobulated margins (9–12). In this study, a solid, indeterminate lesion was defined as a non-calcified lesion or a lesion without features strongly suggestive of a benign etiology, usually greater than 8 mm in size (13).

Recently, machine learning has shown outstanding capabilities as one of the most promising tools for the detection, diagnosis, and differentiation of lung lesions. Over the years, two computational strategies have been developed to predict the malignancy of lung lesions on CT images: radiomics based on quantitative radiological image features and deep learning methods such as those based on cascade convolutional neural networks (CNNs). The extraction of radiomics features relies heavily on accurate lesions boundary outline, and predictive models are built based on a prior knowledge of which features are significant. Whereas CNNs could automatically extract potential features beyond human perception from medical images to predict whether a lesion is benign or malignant by amplifying aspects of the input images that are important for discrimination and suppressing irrelevant variations (14). When successfully applied, it is expected to improve diagnostic accuracy and reduce unnecessary invasive procedures and costs and anxiety of patients. Several studies have revealed the predictive value of CNNs and the promising prospects they afford for lung lesion differentiation (15–18). However, (1) these models lack interpretability and are often referred to as “black boxes”, which renders them difficult for the users to understand; (2) no specific emphasis has been given to distinguishing benign from malignant lesions manifesting as solid, indeterminate pulmonary lesions. Therefore, this study aimed to develop an interpretable CNN-based non-invasive diagnostic model for solid, indeterminate SPNs or SPMs on CT and to evaluate its clinical utility.

## 2 Materials and Methods

The retrospective study was approved by the ethics committee of “Shanghai Pulmonary” Hospital. The informed consent requirement was waived.

### 2.1 Study Population

**Figure 2:**
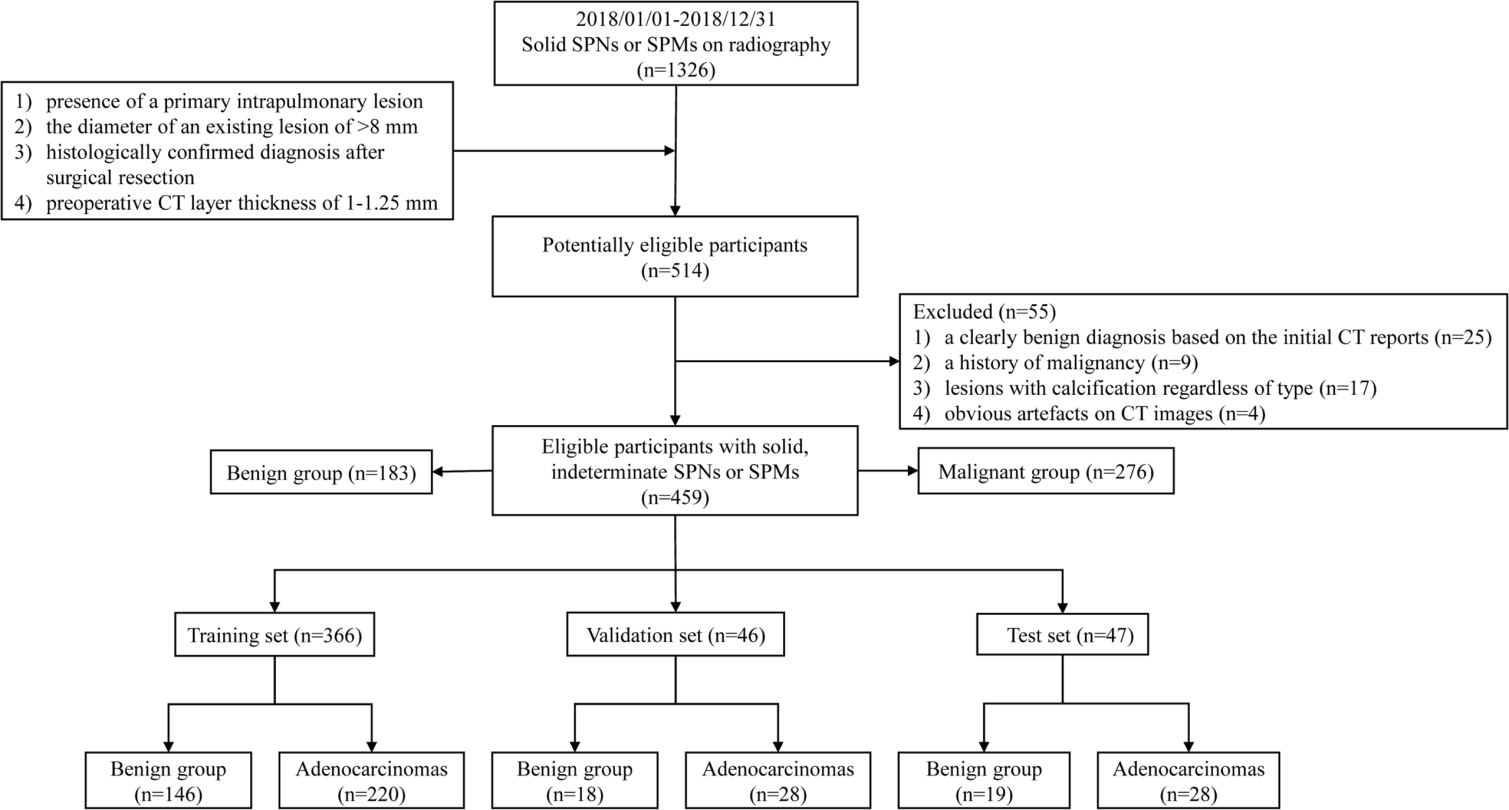
Flow chart of inclusion and exclusion criteria for eligible patients and specific allocations in the train, validation, and test sets.

We retrospectively included 459 consecutive patients with solid, indeterminate SPNs or SPMs on CT between January 2018 and December 2018. Patients who met the following criteria were included: (1) presence of a primary intrapulmonary lesion; (2) the diameter of an existing lesion of >8 mm [because pure-solid nodules measuring <8 mm has the relatively low prevalence of malignancy, and the risks of surgical diagnosis usually outweigh the benefits (13); thus, the Fleischner Society guidelines recommend routine follow-up for management (19), and additionally, in our hospital, one of the criteria for surgical excision is a diameter greater than 8 mm (20)]; (3) histologically confirmed diagnosis after surgical resection; and (4) preoperative CT slice thickness of 1–1.25 mm. The exclusion criteria were as follows: (1) a clearly benign diagnosis based on the initial CT reports; (2) a history of malignancy; (3) lesions with calcification regardless of type; (4) obvious artefacts on CT images. Eligible patients were sorted randomly, and the benign and malignant groups were divided into the training (n=366), validation (n=46), and test (n=47) sets according to the 8:1:1 ratio for model learning, respectively, shown in **Figure 2**.

### 2.2 CT Parameter Acquisition and Image Annotation and Interpretation

All patients underwent Chest CT examinations before surgery in our institution, and the detailed scanning parameters are shown in **Supplement Table 1**. Two thoracic radiologists (with 3 and 7 years of work experience) detected the location of pulmonary lesions, marked their coordinates (X, Y, and Z axes), and measured their diameters on the section that displayed the longest diameter of the lesion. When annotations differed, radiologists discussed them until consensus was achieved.

Additionally, these lesions were evaluated for shape (regular or irregular), the presence of spiculation, lobulation, and pleural retraction. The mean CT density was calculated by measuring the average CT value of the region of interest (ROI) that carefully placed in an area away from vessels, bronchi, and necrosis. The readings were interpreted using Radiant software (http://radiantviewer.com) with the lung window setting (window level, −450 Hounsfield unit [HU]; width, 1,500 HU) and mediastinal window setting (window level, 40 HU; width, 400 HU). Based on the experienced evaluation, the other two radiologists (with 4 and 9 years of experience in reading thoracic CT scans, respectively), uninformed of the pathological results, made a diagnosis. In case of a discrepancy between the two radiologists, a third radiologist with an experience of more than 29 years in thoracic CT made the final decision.

### 2.3 CNN Model Construction

#### 2.3.1 Image and Data Preprocessing

**Figure 3:**
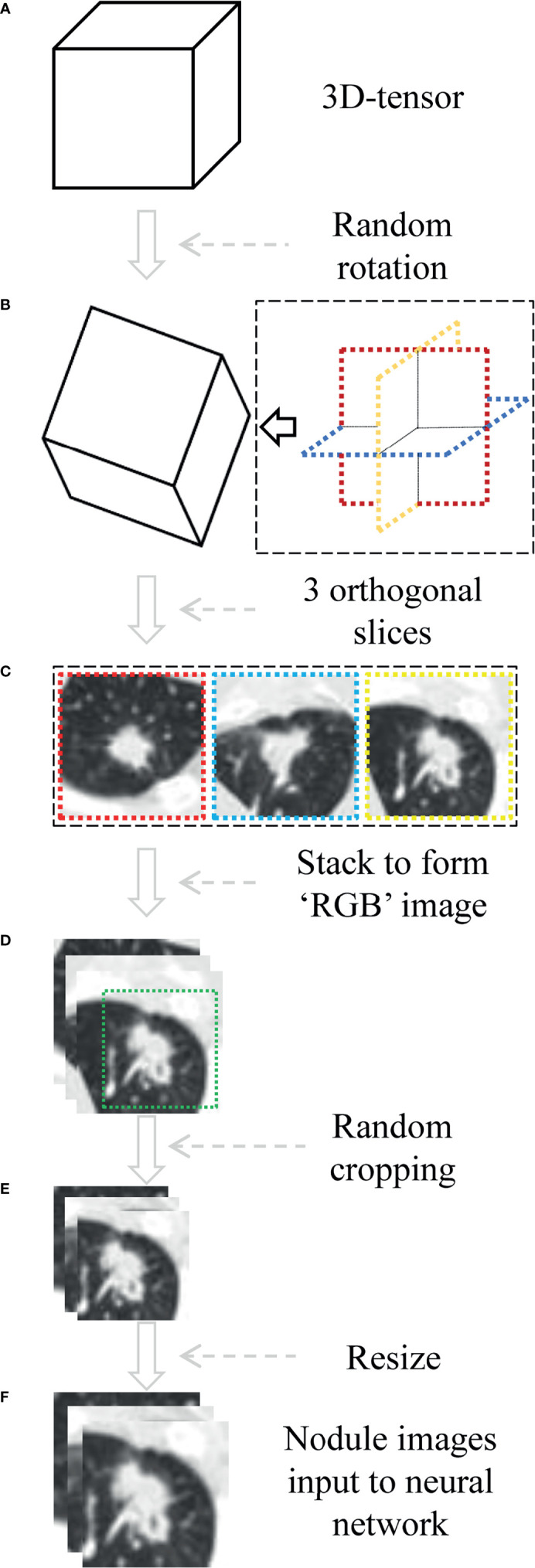
The process of data preprocessing. **(A)** Three-dimensional (3D) tensor was obtained from the original CT sequence according to nodule coordinates labeled by radiologist. **(B)** The tensor is rotated at arbitrary angle around its center point. **(C, D)** Three orthogonal slices spanning center point were extracted and stacked to form a pseudo-RGB map (3×128×128 tensor). **(E)** Random cropping with 3×104×104 subregion. **(F)** Nodule images were resized to voxels of 3×224×224.

The voxel spatial resolution of all patients’ raw CT were standardized on all three axes, to 0.6 × 0.6 × 0.6 mm^3^ each voxel. Then small three-dimensional (3D) tensor with size of 128×128×128 voxels centered at each nodule is extracted using corresponding coordinates annotation. The size ensured that each nodule was entirely covered. In the training phase, it was necessary to randomly rotate the tensor at arbitrary angle in the 3D space, as a data augmentation method. Then we selected three orthogonal slices passing through the center point and stacked them, resulting in a 3×128×128 tensor. Furthermore, we cropped a 3×104×104 sub-region that could completely cover all lesions, and resized it to the voxels of 3×224×224, as a data augmentation method also. Finally, the CT value interval was clipped to [−1,100 HU, 100 HU], and the result was further linearly mapped to the value interval [0, 1]. Each 3×224×224 tensor represented one patient in the network pipeline. The preprocessing was shown in **Figure 3**.

#### 2.3.2 The Structure of the CNN Model

**Figure 4:**
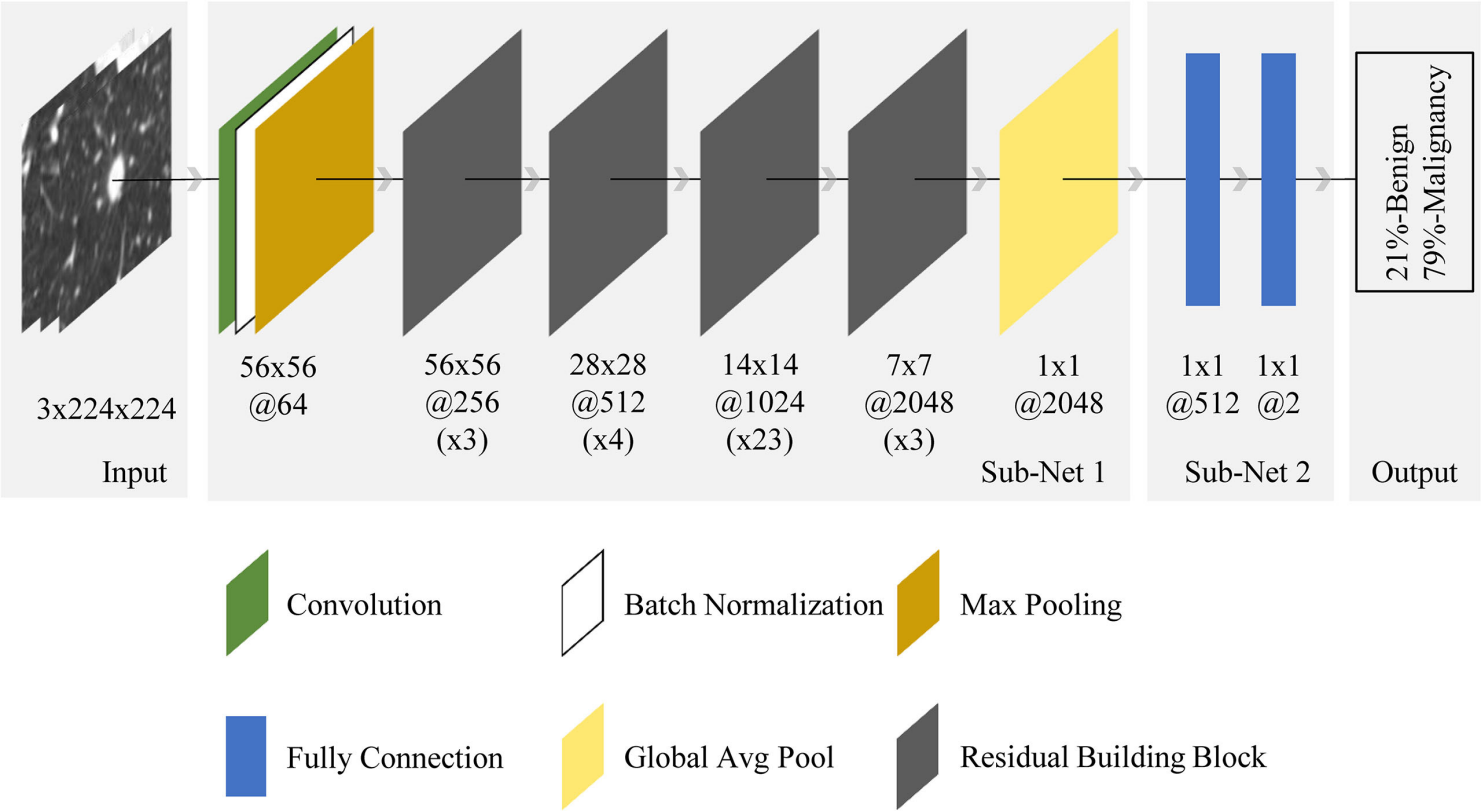
End-to-end CNN model illustration. For the input nodule images (from the left side), the neural network made the prediction (right side) and outputted two values, representing benign and malignant probabilities (summed to 1). The final diagnosis of each nodule by the model depended on which class was predicted with a probability greater than 50%. The architecture was composed of convolution, batch normalization, max pooling, fully connection, global average pooling, and residual building block. Sub-net 1 was pretrained on ImageNet dataset with ~15 million neutral images, while sub-net 2 was trained from scratch. The 56x56@256(x3) below the first residual building block meant the spatial size and number of channels of the output feature map in this block were 56x56 and 256 respectively, while x3 meant the block contained 3 residual units.

**Figure 4** shows the pipeline of benign and malignant prediction for solid, indeterminate SPNs or SPMs on CT. ResNet was used as the basis of the deep learning model (21). Specifically, the selected network was ResNet-101. As a transfer learning method, ResNet’s weights parameter pre-trained on the ImageNet image dataset was loaded to initialize sub-network 1 of our model (22). In sub-network 2, the last two layers in the original network were replaced by two fully connectied layers with 512 and 2 output nodes, respectively, and their weight parameters were initialized randomly. We chose to use 2D-CNN rather 3D-CNN for the following reasons: (1) the number of CT cases is small and training CNNs from scratch on top of this would lead to overfitting, and there are currently no pre-trained 3D-CNN model weights available on large publicly available 3D CT datasets; (2) the data augmentation method used in the paper that rotated in 3D space could also help 2D-CNN capture the 3D features of nodules, and better prediction results could be achieved using 2D-CNN model with transfer learning. Furthermore, to increase the generalizability of the model and avoid overfitting, mix-up algorithm was adopted (23).

#### 2.3.3 Experiment Parameter Setting

The train dataset was used to train the deep learning algorithm, a separate validation dataset to tune parameter, and the test dataset to assess the final model. During the training stage, only weight parameters in the last two fully connected layers and all batch normalization layers in the network were trained for 1 epoch, and others remained unchanged. This can be considered as a warm-up training. Then the entire model was trained for additional 60 epochs. This process simultaneously optimized all network layers, making the lower convolutional layer more suitable for edge and corner features in CT data, as well as for the specific data distribution resulting from our combination of orthogonal slices. The weights corresponding to the epoch with the lowest validation loss were chosen as the optimal model and saved. The model used Adam as weights optimizer and cross-entropy as loss function (24). The learning rate was 1e-2, and weight decay was 5e-5. One cycle strategy was used to adjust the learning rate during model training (25). The dropout probabilities of the last two fully connected layers in the model were set 0.25 and 0.5, respectively. A batch size of 64 was used. It took about 5 s to train the neural network on all 366 training samples (tensor size: 366×3×224×244) for one epoch. See the code for the detailed procedure. Code implementation was based on the fastai framework (26) and available online https://github.com/DrIsDr/TJU_Chen_SK.

#### 2.3.4 Visualization of the CNN Model

**Figure 5:**
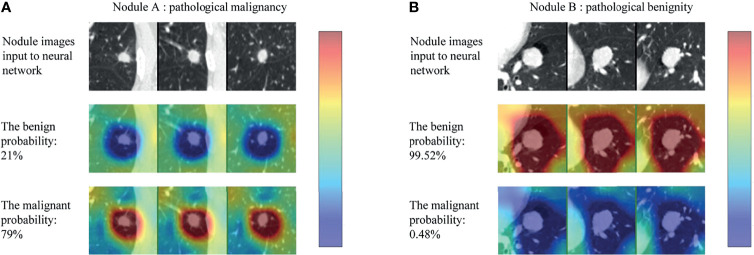
Class activation map (CAM) for two example nodules (nodule **A** and **B**) in the test set. For each nodule, the first row (nodule images input to neural network) represented the three views of each nodule, the second (the benign probability) and third row (the malignant probability) represented the corresponding response heatmaps when the model classifies the nodule as benign and malignant, respectively (red regions are of highest interest and blue lowest).

The CNN models were often referred to “black-box” technology due to lack of interpretability, making it difficult for users to understand the inference procedure. We used the class activation map (CAM) to visualize the discriminative process of the neural network (27), and the results are shown in **Figure 5**. The CAM could generate the response heatmaps to reversely deduce the process of the model making diagnosis. Red areas had the highest activation value, which suggested that the model mainly extracted diagnostic characteristics from the region, whereas the blue areas had the lowest activation value, meaning that less discriminative features were found in this region.

As can be seen in the **Figure 5**, the CNN model produced high activation value (red areas) in the regions where the nodule was located and adjacent to the nodule only when the nodule was correctly classified. In other words, the model captured the internal and external features of nodules to make a diagnosis. More examples are shown in the **Supplementary Figure 1**.

### 2.5 Statistical Analysis

Baseline characteristics and image information of the participants were summarized as mean ± standard deviations (SD) values for continuous variables, and as frequency and percentage for categorical variables. Statistical significance was tested using Student’s t-test, Welch’s t-test, Mann-Whitney U-test, and Kruskal-Wallis for continuous variables as appropriate, and Chi-square test for categorical variables. A p-value < 0.05 was considered to be statistically significant. The predictive results of CNN model were compared with the pathological gold standard. The diagnostic performance of the CNN model was described using the area under the receiver operating characteristic (ROC) curve (AUC) with 95% CI. The mean value of accuracy, sensitivity, and specificity of diagnoses by radiologists alone were compared with those of diagnoses by radiologists with the assistance of the CNN model to evaluate its clinical utility. All statistical analyses are based on SPSS 20.0 software (SPSS Inc., Chicago, IL, USA) and R version 3.6.3 (R foundation for Statistical Computing).

## 3 Results

### 3.1 Baseline Characteristics

A total of 459 patients with solid, indeterminate SPNs or SPMs were included. Of the 459 patients, 183 had benign disease (83 males and 100 females; mean age, 53.67 ± 12.33) and 276 had malignant disease (151 males and 125 females; mean age, 60.53 ± 9.30). Among the 183 benign cases, there were 124, 55, and 4 granulomas, pulmonary sclerosing pneumocytomas (PSPs), and focal organizing pneumonia (FOP), respectively. The subtype of malignancy only included lung adenocarcinomas.

The clinical baseline characteristics and image features are listed in **Table 1**. Between the benign and malignant groups, clinical variables, such as age (P<0.01) and gender (P=0.05), demonstrated statistical difference. However, no statistically significant association was observed in terms of radiological features.

**Table 1:** The baseline characteristics and imaging information of patients included in the study.

Variables	Total (n=459)	Benign (n=183)	Malignant (n=276)	P-value
Age, mean ± SD, y	57.80 ± 11.12	53.67 ± 12.33	60.53 ± 9.30	<0.01
Gender, n (%)				0.05
Male	234 (51)	83 (45)	151 (55)	
Female	225 (49)	100 (55)	125 (45)	
Image information				
Diameter, n (%)				0.14
≤30 mm	379 (83)	157 (86)	222 (80)	
>30 mm	80 (17)	26 (14)	54 (20)	
Tumors location, n (%)				0.07
RUL	123 (27)	40 (22)	83 (30)	
RML	45 (10)	19 (10)	26 (9)	
RLL	97 (21)	50 (27)	47 (17)	
LUL	111 (24)	41 (22)	70 (25)	
LLL	83 (18)	33 (18)	50 (18)	
Shape, n (%)				0.50
Regular	103 (22)	44 (24)	59 (21)	
Irregular	356 (78)	139 (76)	217 (79)	
Lobulation, n (%)				0.59
Presence	290 (63)	117 (64)	173 (63)	
Absence	168 (37)	66 (36)	102 (37)	
Spiculation, n (%)				
Presence	244 (53)	94 (51)	150 (54)	0.53
Absence	215 (47)	89 (49)	126 (46)	
Pleural retraction, n (%)				0.92
Presence	232 (51)	93 (51)	139 (50)	
Absence	227 (49)	90 (49)	137 (50)	
CT value, mean ± SD, HU	31.24 ± 24.86	33.07 ± 27.22	30.03 ± 23.14	0.20

The data are expressesed as mean ± standard deviations for continuous variables, and the frequency and percentage for categorical variables.

A p-value < 0.05 was supported to be statistically significant.

### 3.2 Performance of the CNN Model

**Figure 6:**
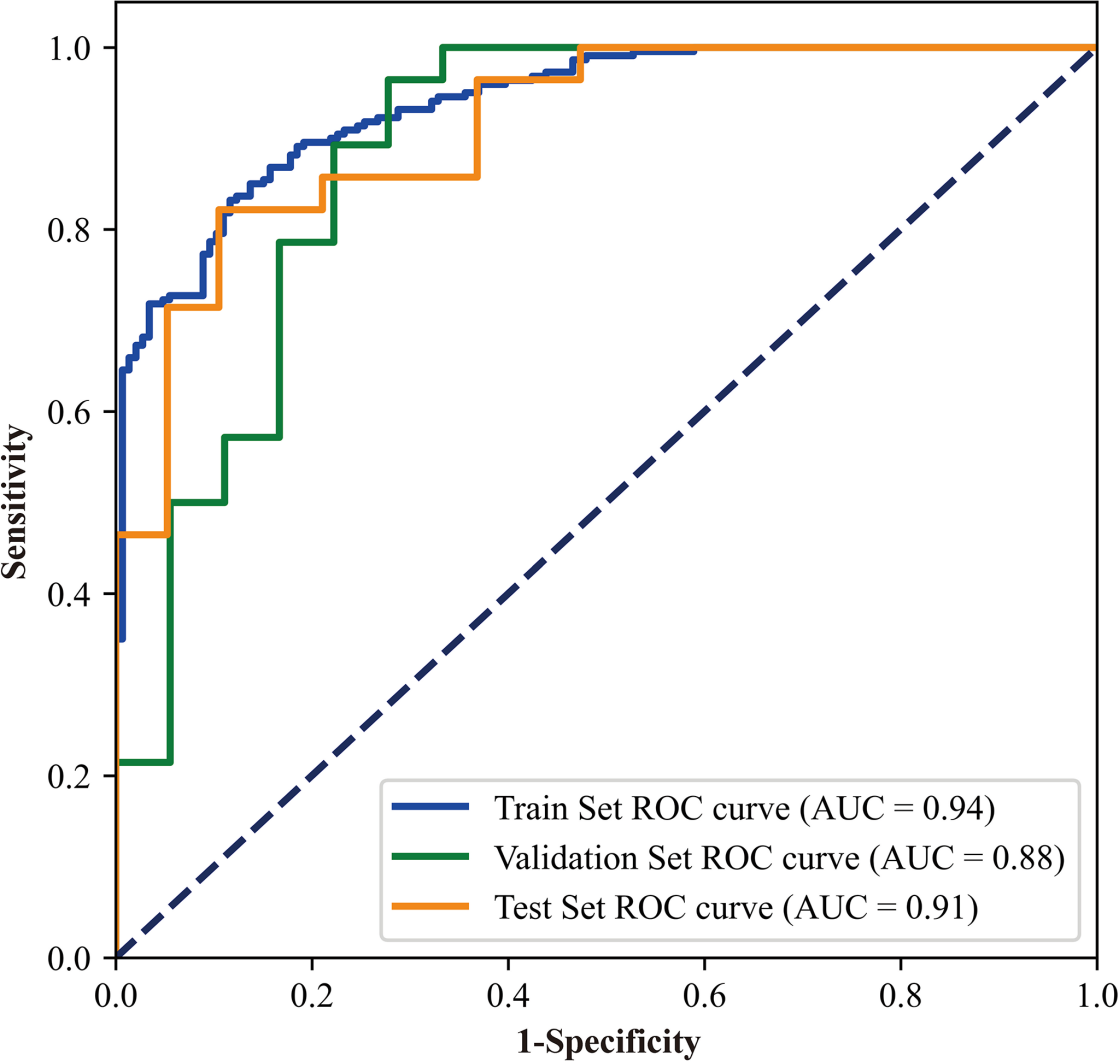
The receiver operating characteristic curves of the CNN model used in this study.

The model demonstrated superior performance in the train set (AUC: 0.94, 95% CI: 0.92–0.96); the results in the validation and test sets showed slightly lower but still satisfactory differentiation performance (validation set: AUC 0.88, 95% CI: 0.78–0.99; test set: AUC 0.91, 95% CI: 0.83–0.99) (**Figure 6**). The total concordance rates between the CNN model and final pathological assessments generated by the final paraffin section in the train, validation, test cohorts were 87% (318/366), 83% (38/46), and 83% (39/47), respectively (**Supplement Table 1**). The sensitivity and specificity were 89% (95% CI: 0.85–0.92) and 84% (95% CI:0.80–0.87) in the train set, 86% (95% CI: 0.73–0.93) and 78% (95% CI: 0.64–0.88) in the validation set, and 86% (95% CI: 0.73–0.93) and 79% (95% CI: 0.65–0.88) in the test set (**Table 2**).

**Table 2:** Predictive performance of the CNN model.

	Train set	Validation set	Test set
AUC	94 (0.92–0.96)	88 (0.78–0.99)	91 (0.83–0.99)
ACC	87 (0.83–0.90)	83 (0.70–0.91)	83 (0.70–0.91)
SE	89 (0.85–0.92)	86 (0.73–0.93)	86 (0.73–0.93)
SP	84 (0.80–0.87)	78 (0.64–0.88)	79 (0.65–0.88)

All values shown as % (95% confidence interval).

CNN, convolutional neural network; AUC: area under curve; ACC, accuracy; SE, sensitivity; SP, specificity.

### 3.3 Clinical Utility of the CNN Model

Three radiologists blinded to the pathological results twice assessed the benignity or malignancy of each patient and made a final decision in consensus. The average time required for diagnosing each patient was 3 min. The diagnostic accuracy of radiologists alone was lower than that of the CNN model in all patients (train set: 66 *vs.* 87%, P<0.01; validation set: 61 *vs.* 83%, P=0.02; test set: 66 *vs.* 83%, P=0.06). When radiologists used the CNN model, their diagnostic accuracy was higher than that achieved by radiologists alone (train set: 89 *vs.* 66%, P<0.01; validation set: 87 *vs.* 61%, P<0.01; test set: 85 *vs.* 66%, P=0.03) (**Supplement Table 2**). Additionally, specificities increased significantly, by an average of 42% (train set: from 43 to 82%; validation set: from 33 to 78%; test set: from 42 to 84%; all P-values < 0.01); and sensitivities improved slightly (train set: 81 *vs.* 95%, P<0.01; validation set: 79 *vs.* 93%, P=0.04; test set: 82 *vs.* 89%, P=0.37) (**Table 3**). Thus, the CNN model could help radiologists to enhance the capability of distinguishing benign from malignant lesions with radiographic solid, indeterminate SPN or SPM characteristics at all three levels of CT expertise, effectively preventing misdiagnosis.

**Table 3:** Comparison of the diagnostic performance of radiologists without and with the CNN model.

	Training set		Validation set		Test set	
	Radiologists alone	Radiologists with CNN	Radiologists alone	Radiologists with CNN	Radiologists alone	Radiologists with CNN
ACC	66 (0.61–0.71)	89 (0.85–0.92)	61 (0.47–0.74)	87 (0.74–0.94)	66 (0.52–0.78)	85 (0.72–0.93)
SE	81 (0.77–0.85)	95 (0.92–0.97)	79 (0.65–0.88)	93 (0.82–0.98)	82 (0.69–0.90)	89 (0.77–0.95)
SP	43 (0.38–0.48)	82 (0.78–0.86)	33 (0.21–0.47)	78 (0.64–0.88)	42 (0.29–0.56)	84 (0.71–0.92)
FPV	57 (0.52–0.62)	19 (0.15–0.23)	67 (0.53–0.79)	22 (0.12–0.36)	58 (0.44–0.71)	16 (0.08–0.29)
FNV	19 (0.15–0.23)	6 (0.04–0.09)	21 (0.12–0.35)	7 (0.02–0.18)	18 (0.10–0.31)	11 (0.05–0.23)

All values shown as % (95% confidence interval).

CNN, convolutional neural network; ACC, accuracy; SE, sensitivity; SP, specificity; FPV, false positive value; FNV, false negative value.

## 4 Discussion

Deep CNN is a type of deep learning approach in which computers are not explicitly programmed but can perform tasks by analyzing relationships of existing data. In this retrospective study, our CNN model achieved better accuracy than three radiologists in differentiation between benignity and malignancy for solid, indeterminate SPNs or SPMs. When radiologists used the CNN model, the mean accuracy was 87%, and the specificity improved by 42%, which would have facilitated timely diagnosis and treatment for lung cancer and avoided unnecessary excision for benign cases by a non-invasive, highly efficient, and reproducible method. Furthermore, to enhance interpretability, we used visualization techniques to analyze the process of the CNN model classification. To the best of our knowledge, this is the first attempt to differentiate radiographically solid, indeterminate lesions using interpretable CNN technology based on thin-section CT scans.

The solid SPN is an extremely common type of tumor, and approximately 60% of solid SPNs are malignant (1, 2). Published studies have reported that nodal metastasis and intrapulmonary and extrapulmonary diffusion could be found in malignant solid lesions, even in subcentimeter small nodules (4). Thus, differentiation of benign and malignant lesions is the most critical step for patient management.

Chest CT examinations can provide specific information about morphological and density characteristics and are helpful to estimate the probability of malignancy for pulmonary solid lesions. Multiple studies have revealed that spiculation, lobulation, irregular shape, and pleural retraction are associated with malignancy, whereas lesions with a regular shape and the smooth margin are more likely to be benign (28, 29). However, in our dataset, no radiologically available features were observed (**Table 1**), which means that trained radiologists have difficulty distinguishing the nature history of solid, indeterminate solitary pulmonary by visual assessment alone. The overlap of radiographic characteristics does not seem too unusual. As noted previously, for pulmonary lesions with smooth edges, the risk of malignancy was approximately 35% (range: 21–58%) (9–12). Chu et al. reported that 95% (214/225) of solid cancerous nodule had a regular shape (30). Also, Zerhouni et al. recorded that 25% of benign nodules showed irregular margins with lobulation or spiculation, and only 18% of these lesions were correctly assessed on CT (9). In addition, in the study by Xu et at., lung cancer risk was absent in solid indeterminate nodules attached to the pleural or a fissure during 1 year of follow-up (31).

Thus, it is very pivotal to differentiate benign from malignant solid, indeterminate SPNs or SPMs by using a new approach to overcome the naked limitation. However, some studies revealed and exploited the massive potential of image features that may be visually imperceptible to even very experienced thoracic radiologists and can be extracted from CT scans by using (1) radiomics methods or (2) deep learning approaches based on CNNs (32–34). Both methods have been widely used to classify and identify the natural history of sub-solid nodules including the part-solid and pure ground glass nodules, scoring tremendous achievements (35–39). Nevertheless, few studies have focused on the differentiation of solid pulmonary lesions. Shen et al. established a multiclassifier fusion based on radiomic features, including geometric features, textures features, gray-level features, and wavelet features to predict benign and malignant primary solid nodules, achieving an AUC of 0.915 in the test set (40). However, not all benign cases in this study were pathologically confirmed, a stable 2-year follow-up period does not guarantee its benign nature. In addition, radiomics methods that extract quantitative biological features are limited by prior knowledge of significant characteristics, which may be unbefitting for pulmonary lesions with considerable overlapping features. The CNN method could simplify the redundancies and learn discriminating features directly from CT images, facilitating greater reproducibility. In this study, our CNN model in the test set had an AUC of 0.91, comparable with the previously reported value, which indicated good performance. The specificity of our model was significantly higher than that of the three radiologists. In fact, most benign lesions in our study mimicked the morphological characteristics of lung cancer. Radiologists are prone to classifying these lesions as malignant in clinical practice, yielding high sensitivity with low specificity. However, when radiologists used the CNN model, their specificity improved significantly by 42% while maintaining the high sensitivity.

Additionally, the CNN model is highly efficient in distinguishing benignity from malignancy for solid, indeterminate lesions. Radiologists spent an average of 3 min to read and interpret a set of CT images of one patient, while the CNN model could process the 366 patient images in just 5 s. Moreover, in routine clinical practice, radiologists usually need to review and compare prior CT images to make a diagnosis, which would require more time despite yielding higher accuracy. In our model, on the basis of coordinate information, we adopted a supervised learning method, guided the neural network model to extract features layer-by-layer from CT images of interest, constantly enhanced the intensity of feature abstraction, and finally output the result of the prediction. Thus, we used an end-to-end computational method that could greatly simplify the traditional workflow.

A limitation of our model is the overfitting issue caused by the single-institute small data size. To compensate for this limitation, we utilized the pretrained network on ImageNet that included millions of natural images, in a process termed transfer learning. Although there is no intuitive approach for using a pretrained model with non-medical images for differentiation of medical images, some features including the edges, corners, orientations, and textures are generic. We compared the performance of the CNN model with or without pretrained procedure using the same experimental parameters, and the results showed the pretrained CNN model performed much better than the untrained one (**Supplementary Figure 2**). In addition, data augmentation was also used to resolve this problem. Randomly rotating nodules/masses in 3D space, extracting three orthogonal slices to form pseudo-RGB map, cropping, and resizing the input images for neural network could help 2D-CNN capture rich 3D features. In the future, a larger multicenter study should be used to validate this model and improve the performance of this algorithm.

Other limitations should be mentioned. Lesions were not automatically detected, but based on radiologist annotations, which would lead to interobserver variability and error propagation to CNN model because of this process. However, to reduce this bias, all lesions were marked in consensus by two experienced radiologists. Furthermore, we used the Grad-CAM method to visualize the intermediate variables generated by the trained model for the prediction process of the images. Given an image patch, the model does focus on the nodule, demonstrating that the region of interest used by the model for feature recognition is correct and that such interpretable analysis is appropriate for the form of our annotation (which includes nodule location and class) currently provided. At present, the design of CNN algorithms and the abundance of clinical data are mutually reinforcing. In the future, as more data become available and finer-level annotation information becomes more widespread, CNNs can be more useful for clinical applications. Considering actual clinical limitations, the design of our cohort was restricted to allow differentiation between adenocarcinomas and benign diseases including granulomas, PSP, and FOP. Actually, it makes sense to use CNN model to further predict the results of benign lesions for subclassification. However, in our study, we did not perform this task. Understandably, the multi-classification tasks for benign lesions are difficult due to the disparity in sample distribution of benign subtypes (124 granulomas; 55 PSPs; 4 FOPs), as a well-performing model requires a large number of sample data of each type of disease. We plan to conduct a more in-depth evaluation of the application of CNN model to multi-classification tasks based on large samples in the upcoming studies.

In conclusion, we established a CNN model based on CT images that can serve as a valuable tool for radiologists to differentiate radiographic solid, indeterminate SPNs or SPMs. Moreover, a visualization procedure was presented to enhance interpretability of CNN model.

## Data Availability Statement

The datasets presented in this study can be found in online repositories. The names of the repository/repositories and accession number(s) can be found below: https://github.com/DrIsDr/TJU_Chen_SK.

## Author Contributions

KS: Conception and design, data analysis and interpretation, manuscript writing, final approval of manuscript. SC: Data analysis and interpretation, manuscript writing, final approval of manuscript. JZ: Collection and assembly of data, manuscript writing, final approval of manuscript. BW: Manuscript writing, final approval of manuscript. YY: Manuscript writing, final approval of manuscript. YW: Data analysis and interpretation, manuscript writing, final approval of manuscript. CW: Provision of study materials or patients, manuscript writing, final approval of manuscript. XS: Conception and design, administrative support, manuscript writing, final approval of manuscript. All authors contributed to the article and approved the submitted version.

## Funding

This work was supported by the Natural Science Foundation of Shanghai (grant Number 21Y11910400). The funders had no role in the study design, data collection, data analysis, and writing the manuscripts.

## Conflict of Interest

The authors declare that the research was conducted in the absence of any commercial or financial relationships that could be construed as a potential conflict of interest.

## Publisher’s Note

All claims expressed in this article are solely those of the authors and do not necessarily represent those of their affiliated organizations, or those of the publisher, the editors and the reviewers. Any product that may be evaluated in this article, or claim that may be made by its manufacturer, is not guaranteed or endorsed by the publisher.

## Supplementary Material

Supplementary Figure 1 The more represented results for using CAM to visualize the discriminative process of the neural network. (nodules **A–D**): examples of CNN model incorrectly predicting the benignity and malignancy of nodules. (nodules **E–J**) examples of CNN model for accurate prediction of nodal benignity and malignancy. As shown in nodule H, the ROI heat values used to determine malignancy is low, but the ROI heat values used to determine non-benign is high, which can be explained by the fact the CNN determines this case as malignant by referring more to the surrounding area than to the nodal region. What is more, we can learn from nodule I that the CT images of this case obviously have texture noise that is not present in other cases, but the CNN still correctly detects the ROI and makes a judgment, which reflects the robustness of the CNN model. CAM, class activation map; CNN, convolutional neural network; ROI, region of interest; CT, computed tomography.

## Abbreviations

CT, computed tomography; SPNs, solitary pulmonary nodules; SPMs, solitary pulmonary masses; CI, confidence interval; HRCT, high-resolution computed tomography; CNN, cascade convolutional neural network; ROI, region of interest; HU, Hounsfield unit; 3D, three-dimensional; CAM, class activation map; SD, standard deviations; ROC, receiver operating characteristic; AUC, area under the ROC; PSPs, pulmonary sclerosing pneumocytomas; FOP, focal organizing pneumonia.
