# Corrigendum: Convolutional neural network-based diagnostic model for a solid, indeterminate solitary pulmonary nodule or mass on computed tomography

**DOI:** 10.3389/fonc.2023.1302777

**Published:** 2023-11-07

**Authors:** Ke Sun, Shouyu Chen, Jiabi Zhao, Bin Wang, Yang Yang, Yin Wang, Chunyan Wu, Xiwen Sun

**Affiliations:** ^1^ Department of Radiology, Huashan Hospital, Fudan University, Shanghai, China; ^2^ Department of Radiology, Shanghai Pulmonary Hospital, Tongji University School of Medicine, Shanghai, China; ^3^ Department of Computer Science and Technology, College of Electronics and Information Engineering, Tongji University, Shanghai, China; ^4^ Department of Pathology, Shanghai Pulmonary Hospital, Tongji University School of Medicine, Shanghai, China

**Keywords:** neural network model, computed tomography, differential diagnosis, solid, indeterminate solitary pulmonary nodule, lung adenocarcinoma

In the published article, there was an error in **Figure 1** as published. **Figure 1D2** and **Figure 1C2** were duplicated by mistake. The corrected **Figure 1** and its caption appear below.

**Figure 1:**
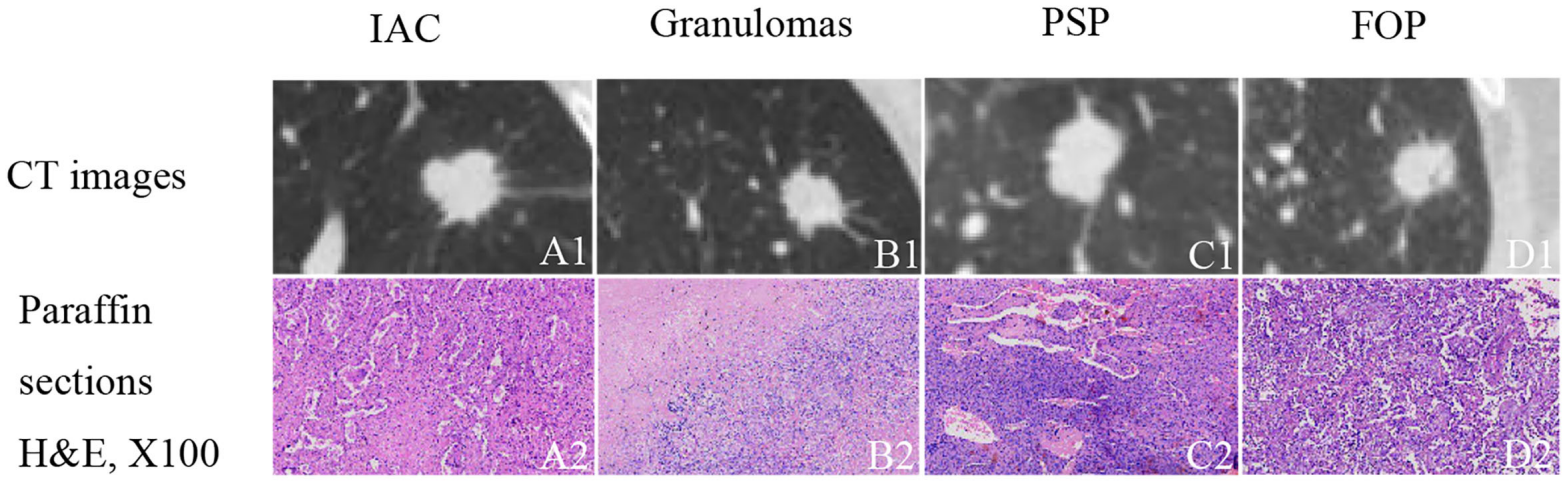
Examples of solid, indeterminate SPN/SPMs without features strongly suggestive of a benign etiology. **(A1)** Invasive adenocarcinoma (IAC); **(B1)** granuloma; **(C1)** pulmonary sclerosing pneumocytoma (PSP); **(D1)** focal organizing pneumonia (FOP). **(A2–D2)** paraffin section (hematoxylin and eosin [H&E], 100 ×) of IAC, granuloma, PSP, and FOP, respectively.

The authors apologize for this error and state that this does not change the scientific conclusions of the article in any way. The original article has been updated.

