# Supplementary material for: Convolutional Neural Network-Based Diagnostic Model for a Solid, Indeterminate Solitary Pulmonary Nodule or Mass on Computed Tomography
Source: Front Oncol. 2021 Dec 21;11:792062. doi: 10.3389/fonc.2021.792062 (PMC8724915; doi:10.3389/fonc.2021.792062)
Supplement: Supplementary file 2 [file DataSheet_2.pdf]

main_with_imagenet

November 13, 2021

1 Convolutional Neural Network-based Interpretable Diagnostic Model for A Solid, Indeterminate Solitary Pulmonary Nodule or Mass on Computed Tomography

[1]:
```
import fastai
from fastai.torch_core import defaults
from fastai.vision.all import doc, DataBlock, ImageBlock, CategoryBlock,␣
 ↪GrandparentSplitter,get_files, \
FileGetter,parent_label,to_image,PILBase,Image,ToTensor,TransformBlock,IntToFloatTensor,PILIma
Resize,Normalize,imagenet_stats,Learner,resnet34,CrossEntropyLossFlat,accuracy,RocAucBinary,PI
TensorImageBase,xresnet34,Module,model_meta,create_body,create_head,params,xse_resnext101,xse_
xse_resnext18,aug_transforms,RandomResizedCrop,RocAuc,Recorder,ImageDataLoaders,Interpretation
xresnet18,xresnet50,xresnet101,xresnet152,skm_to_fastai,xse_resnet152,RandomCrop,MultiCategory
get_files,TfmdLists,show_at,Datasets,Categorize,resnet50,resnet101,resnet152,cast,store_attr,f
ClassificationInterpretation,get_grid,CropPad,Resize,ResizeMethod,Pipeline,uniqueify,detuplify
from fastai.callback.mixup import MixUp
from fastai.callback.tracker import SaveModelCallback
from fastai.data.core import tensor, Tensor, show_image, fastuple,␣
 ↪explode_types, typedispatch, DataLoaders
from fastcore.foundation import L
from fastai.torch_basics import torch, nn, set_seed, is_listy, flatten_check,␣
 ↪to_np, itertools
from fastai.optimizer import SGD, Adam
from multiprocessing import Pool
from torch import nn
from torch.nn import functional as F
import torchvision.models as models
import os
import cv2
from scipy import ndimage
import SimpleITK
import SimpleITK as sitk
from glob import glob
import nibabel as nib
import numpy as np
import cupy as cp
```

```python
from cupyx.scipy import ndimage as cu_ndimage
import random
from termcolor import colored
from pathlib import Path
from PIL import Image
import PIL
import pandas as pd
import shutil
from tqdm import tqdm_notebook
import sklearn.metrics as skm
from functools import partial
import copy
import matplotlib
import matplotlib.pyplot as plt

plt.rc('font', family="Times New Roman")
%reload_ext autoreload
%autoreload 2
%matplotlib inline

# from IPython.core.interactiveshell import InteractiveShell
# InteractiveShell.ast_node_interactivity = "all"
```

2 Config & Args

[2]:
```python
def random_seed(seed_value):
    """
    Global random seed for CPU/GPU.
    """
    assert isinstance(seed_value, int)

    random.seed(seed_value)  # Python
    np.random.seed(seed_value)  # cpu vars
    torch.manual_seed(seed_value)  # cpu  vars

    if torch.cuda.is_available():
        torch.cuda.manual_seed(seed_value)
        torch.cuda.manual_seed_all(seed_value)  # gpu vars
        torch.backends.cudnn.deterministic = True
        torch.backends.cudnn.benchmark = False


class Args():
    exp_name = 'SK1028'
    seed = 2
    dpi = 800
```

```python
    ww = 1200
    wl = -500
    # /home/user/BP2T/Projects/data/SK
    # excel_path = Path('/Projects/data/SK/Excels')
    # benign_ct_path = Path('/Projects/data/SK/2018  ')
    # malignant_ct_path = Path('/Projects/data/SK/2018   ')
    # output_path = Path('/Projects/data/SK/processed_ct')
    excel_path = Path('/home/user/BP2T/Projects/data/SK/Excels')
    benign_ct_path = Path('/home/user/BP2T/Projects/data/SK/2018  ')
    malignant_ct_path = Path('/home/user/BP2T/Projects/data/SK/2018   ')
    output_path = Path('/home/user/BP2T/Projects/data/SK/processed_ct')
    blacklist = [
        '9001159900',
    ]
    spacing = [0.6, 0.6, 0.6]
    fixed_half_edge = 64  #half the length of nodule 3D-Patch
    use_existing = False
    split_p = (0.8, 0.9)  #Split points for training, validation, and test sets
    radius_middle = 56  #half the length of crop
    resize_size = 224  #size of network's input
    n_in = 3  #number of input channel

    batch_size =16 # 64
    size = 128

    num_workers = 0  #Number of CPU threads in DataLoader
    ps = 0.5
    lr = 1e-2
    wd = 5e-5
    mom = 0.9
    epoch =  60
    exp = '1113'
    pct_start = 0.3
    loss_weight = [1., 1.]

    test_code = False
    pre_processing = False
    plot_validate_result = True


args = Args()
random_seed(args.seed)

defaults.callbacks[1] = Recorder(train_metrics=True)
defaults.callbacks
```

```
[2]: [fastai.callback.core.TrainEvalCallback,
      Recorder,
      fastai.callback.progress.ProgressCallback]
```

2.1 Packages' Version

```
[3]: f'fastai: {fastai.__version__}'
     f'pytorch: {torch.__version__}'
     f'numpy: {np.__version__}'
     f'cupy: {cp.__version__}'
     f'pandas: {pd.__version__}'
     f'matplotlib: {matplotlib.__version__}'
     f'opencv: {cv2.__version__}'
```

```
[3]: 'opencv: 4.2.0'
```

3 Pre-Processing

```
[4]: def load_scan_dcm(path: Path) -> list:
         """
         Tested 10.28

         Read a patient's entire *.dcm file.

         :param path: The path to store a patient's entire *.dcm files.

         :return: The path to store a patient's entire dcm file. list
         """
         assert isinstance(path, Path) and path.is_dir()

         slices = [sitk.ReadImage(str(one_dcm)) for one_dcm in path.glob('*.dcm')]
         slices.sort(
             key=lambda x: x.GetOrigin()[2])  # Sorted on the Depth axis. WHD

         assert len(slices) > 0, str(path) + 'Data is empty'
         assert isinstance(slices[0], SimpleITK.SimpleITK.Image)

         return slices
```

```
[5]: def get_hu_pixels_and_spacing(slices: list) -> tuple:
         """
         Tested 10.28

         Acquire all CT images (DHW) and SPACING parameters for a patient

         :param slices: One patient's entire *.dcm file. list
```

```
    :return: All CT images of a patient in numpy array format (int16, HU units,␣
↪DHW), and Spacing parameters (dimensional order DHW).
    """
    assert isinstance(slices, list)

    image = np.stack(
        [sitk.GetArrayFromImage(s).squeeze() for s in slices],
        axis=0,
    )
    image = image.astype(np.int16)
    spacing = np.array(list(reversed(slices[0].GetSpacing())))

    assert image.dtype == np.int16
    assert image.ndim == 3 and image.shape >= (1, 1, 1), 'dimensional error'
    assert spacing.shape == (3, ), 'Spacing was mishandled.'

    return image, spacing
```

[6]:
```
def re_sample(
    ct_array: np.ndarray,
    x: [int, float],
    y: [int, float],
    z: [int, float],
    r: [int, float],
    spacing: np.ndarray,
    new_spacing: np.ndarray,
    order: int = 3,
) -> tuple:
    """
    Tested 10.28

    Use the new_spacing parameter to resample the voxels in the initial CT␣
↪matrix with spatial resolution, while mapping coordinates, dimensions, and␣
↪other parameters to the pixel coordinate system at the new resolution

    :param ct_array: CT matrix before resampling (dimensional order should read␣
↪DHW)
    :param x: X-coordinate of the nodule centroid
    :param y: Y-coordinate of the nodule centroid
    :param z: Z-coordinate of the nodule centroid
    :param r: Maximum radius length of nodules
    :param spacing: Initial Spacing parameter of the CT matrix
    :param new_spacing: Target Spacing parameter of the CT matrix
    :param order: The order of the interpolation algorithm.

    :return: Resampled CT matrix, true_spacing, x, y, z, length_mm
```

```python
    """
    assert isinstance(ct_array, np.ndarray) and ct_array.ndim == 3
    assert ct_array.shape > (1, 1, 1) and ct_array.dtype == np.int16
    assert isinstance(x, (int, float)) and x >= 0
    assert isinstance(y, (int, float)) and y >= 0
    assert isinstance(z, (int, float)) and z >= 0
    assert isinstance(r, (int, float)) and r > 0
    assert isinstance(spacing, np.ndarray) and spacing.shape == (3, )
    assert isinstance(new_spacing, np.ndarray) and new_spacing.shape == (3, )
    assert isinstance(order, int) and 0 <= order <= 5
    assert (spacing > 0).all() and (new_spacing > 0).all()

    new_shape = np.round(ct_array.shape * spacing / new_spacing)
    new_spacing = ct_array.shape * spacing / new_shape
    resize_factor = new_shape / ct_array.shape

    ct_array = ndimage.interpolation.zoom(
        ct_array,
        resize_factor,
        mode='nearest',
        order=order,
    )
    z, y, x = resize_factor * [z, y, x]
    r *= resize_factor[2]

    assert isinstance(ct_array, np.ndarray) and ct_array.ndim == 3
    assert ct_array.shape > (1, 1, 1) and ct_array.dtype == np.int16
    assert isinstance(new_spacing, np.ndarray) and new_spacing.shape == (3, )
    assert isinstance(x, (int, float)) and x >= 0
    assert isinstance(y, (int, float)) and y >= 0
    assert isinstance(z, (int, float)) and z >= 0
    assert isinstance(r, (int, float)) and r > 0

    return ct_array, new_spacing, x, y, z, r
```

[7]:
```python
def get_nodule(
    ct_array: np.ndarray,
    x: [int, float],
    y: [int, float],
    z: [int, float],
    r: [int, float] = None,
    fixed_half_edge: int = None,
) -> np.ndarray:
    """
    Tested 10.26
```

```
    Based on the label information of the nodule location, a 3D-Patch centered␣
↪on the nodule mass center was intercepted from a CT image of a case.

    :param ct_array: (voxel resolution normalized) numpy matrix (DHW)␣
↪corresponding to a patient's complete CT images
    :param x: X-coordinate of the nodule centroid (after voxel resolution␣
↪normalization)
    :param y: Y-coordinate of the nodule centroid (after voxel resolution␣
↪normalization)
    :param z: Z-coordinate of the nodule centroid (after voxel resolution␣
↪normalization)
    :param r: (voxel resolution normalized) radius of nodule
    :param fixed_half_edge: half length of the desired extracted (voxel␣
↪resolution normalized) cube (in voxels)

    :return: This patient's nodule 3D patch.
    """
    assert isinstance(ct_array, np.ndarray) and ct_array.ndim == 3
    assert ct_array.dtype == np.int16 and ct_array.shape > (1, 1, 1)
    assert isinstance(x, (int, float)) and x >= 0
    assert isinstance(y, (int, float)) and y >= 0
    assert isinstance(z, (int, float)) and z >= 0
    assert isinstance(r, (int, float)) and z >= 0
    assert isinstance(fixed_half_edge, int) and fixed_half_edge > 0

    x, y, z, r = int(x), int(y), int(z), int(r)

    ct_array = ct_array[::-1, :, :]  # reverse D(Z) axis

    ct_array = np.pad(
        ct_array,
        pad_width=fixed_half_edge,
        mode='constant',
        constant_values=ct_array.min(),
    )

    ct_array = ct_array[z:z + fixed_half_edge * 2, y:y + fixed_half_edge * 2,
                        x:x + fixed_half_edge * 2]
    assert ct_array.shape == (
        fixed_half_edge * 2,
        fixed_half_edge * 2,
        fixed_half_edge * 2,
    ), 'Abnormal Nodule Size' + ct_array.shape
    assert ct_array.dtype == np.int16

    return ct_array
```

[8]:
```python
def process_one_case(
    case_path: Path,
    x: [float, int],
    y: [float, int],
    z: [float, int],
    r: [float, int],
    fixed_half_edge: int,
    new_spacing: list,
) -> np.ndarray:  # int16
    """
    Tested 10.28

    Preprocess the CT data from one patient and output the extracted nodule␣
 ↪matrix.


    :param case_path: The path of the folder where the patient's CT images are␣
 ↪stored.
    :param x: X-coordinate of the nodule centroid (after voxel resolution␣
 ↪normalization)
    :param y: Y-coordinate of the nodule centroid (after voxel resolution␣
 ↪normalization)
    :param z: Z-coordinate of the nodule centroid (after voxel resolution␣
 ↪normalization)
    :param r: (voxel resolution normalized) radius of nodule
    :param fixed_half_edge: half length of the desired extracted (voxel␣
 ↪resolution normalized) cube (in voxels)
    :param new_spacing: Target Spacing

    :return: Patient's nodule 3D matrix (int16) (not processed by WL and WW)
    """
    assert isinstance(case_path, Path) and case_path.is_dir()
    assert isinstance(x, (int, float)) and x >= 0
    assert isinstance(y, (int, float)) and y >= 0
    assert isinstance(z, (int, float)) and z >= 0
    assert isinstance(r, (int, float)) and r >= 0
    assert isinstance(fixed_half_edge, int) and fixed_half_edge > 0
    assert isinstance(new_spacing, list) and len(new_spacing) == 3

    slices = load_scan_dcm(path=case_path)
    ct_array, spacing = get_hu_pixels_and_spacing(slices=slices)
    ct_array, true_spacing, x, y, z, r = re_sample(
        ct_array,
        x=x,
        y=y,
        z=z,
        r=r,
```

```
        spacing=spacing,
        new_spacing=np.array(new_spacing),
    )

    patch_3d = get_nodule(
        ct_array,
        x=x,
        y=y,
        z=z,
        r=r,
        fixed_half_edge=fixed_half_edge,
    )

    assert patch_3d.shape == (fixed_half_edge * 2, fixed_half_edge * 2,
                              fixed_half_edge * 2)
    assert patch_3d.dtype == np.int16
    return patch_3d
```

[9]:
```
def worker_for_one_case(
    para: dict,
    processed_path: Path,
    category_names: list,
    fixed_half_edge: int,
    spacing: list,
) -> None:
    """
    Tested 10.26

    Worker for extracting a nodule from a patient and saving the *.npy file to␣
↪the hard drive.

    :param para: parameter information of dictionary type that holds parameter␣
↪related to a patient.
    :param processed_path: The path where the results are stored.
    :param category_names: A list of category name strings.
    :param fixed_half_edge: the number of pixels of fixed half length of the␣
↪nodule to be cropped
    :param spacing: The resolution of each case voxel needs to be normalized to␣
↪this spacing parameter.

    :return: None
    """
    assert isinstance(para, dict)
    assert isinstance(processed_path, Path) and processed_path.is_dir()
    assert isinstance(category_names, list)
    assert isinstance(fixed_half_edge, int) and fixed_half_edge > 0
    assert isinstance(spacing, list) and len(spacing) == 3
```

```
    assert (np.array(spacing) > 0).all()

    npy_folder = processed_path / para['STAGE'] / category_names[para['C']]
    npy_file = npy_folder / f'{para["ID"]}.npy'

    if not npy_folder.exists():
        npy_folder.mkdir(parents=True, exist_ok=True)

    if args.use_existing and npy_file.exists():
        print(f'file {npy_file} existing, skip and do nothing.')
    else:
        patch_3d = process_one_case(
            case_path=para['PATH'],
            x=para['X'],
            y=para['Y'],
            z=para['Z'],
            r=para['R'],
            fixed_half_edge=fixed_half_edge,
            new_spacing=spacing,
        )

        assert patch_3d.dtype == np.int16

        np.save(str(npy_file), patch_3d)
        print(f'file {npy_file} saved')
```

[10]:
```
def get_label_from_pd(
        cases_pd: pd.DataFrame,
        c: int,
        percents: tuple = (0.8, 0.9),
) -> list:
    """
    Tested 10.28

    Extracting label information from benign/malignant DataFrame objects

    :param cases_pd: benign/malignant DataFrame object
    :param c: category identifier, 0: benign; 1: malignant
    :param percents: train/valid/test data split scale points.

    :return: label information
    """
    assert isinstance(cases_pd, pd.DataFrame)
    assert isinstance(c, int) and c in (0, 1)
    assert isinstance(percents, tuple) and len(percents) == 2
    assert isinstance(percents[0], float) and isinstance(percents[1], float)
    assert 0 < percents[0] < percents[1] < 1
```

```
    r = [
        {
            'ID': str(item['  ']),
            'X': int(item['  '].split('.')[0]),
            'Y': int(item['  '].split('.')[1]),
            'Z': int(item['  '].split('.')[2]),
            'C': c,  #category
            'R': float(item[' mm'] / 2),  # nodule radius
        } for index, item in cases_pd.iterrows()
    ]

    count = len(r)

    random.shuffle(r)
    processed = 0
    for i in r:
        if 0 <= processed < int(count * percents[0]):
            i['STAGE'] = 'train'
        elif int(count * percents[0]) <= processed < int(count * percents[1]):
            i['STAGE'] = 'valid'
        elif int(count * percents[1]) <= processed < count:
            i['STAGE'] = 'test'
        else:
            raise ValueError
        processed += 1

    assert len(r) > 0
    return r


if args.pre_processing and args.test_code:
    benign_pd = pd.concat(
        [
            pd.read_excel(
                args.excel_path / 'SK_1 -psp.xlsx')[['  ', '  ', ' mm']],
            pd.read_excel(
                args.excel_path / 'SK_1 -   .xlsx')[['  ', '  ', ' mm']],
            pd.read_excel(
                args.excel_path / 'SK_1 -   .xlsx')[['  ', '  ', ' mm']],
        ],
        axis=0,
        sort=False,
    )
    label_lst = get_label_from_pd(cases_pd=benign_pd, c=0)

    assert isinstance(label_lst, list)
```

```
    assert isinstance(label_lst[0], dict)
    for i in label_lst[:5]:
        print(i)
```

[11]:
```
def get_filtted_case_path_and_label(
    ct_root_path: Path,
    label_info_lst: list,
) -> list:
    """
    Tested 10.26

    Get the paths of all the benign/malignant cases, and
    according to the label list, filter out the cases
    that exist in the corresponding folders, and output
    them in a form that can be used by the nodule extraction
    function.

    :param ct_root_path: benign/malignant CT root directory
    :param label_info_lst: the label list of all benign/malignant cases.
    :return: List of all benign/malignant cases that exist in the corresponding␣
↪folder
    """
    assert isinstance(ct_root_path, Path) and ct_root_path.is_dir()
    assert isinstance(label_info_lst, list) and len(label_info_lst) > 0

    sub_class_paths = [
        ct_root_path / sub_dir for sub_dir in ct_root_path.iterdir()
        if sub_dir.is_dir()
    ]

    cases_path_lst = []
    for sub_class_path in sub_class_paths:
        for sub_class_case in sub_class_path.iterdir():
            if sub_class_case.is_dir():
                cases_path_lst.append(sub_class_case)

    valid_lst = []
    for label_info in label_info_lst:
        if label_info['ID'] in args.blacklist:
            print(
                colored('case {} in black, skip.'.format(label_info['ID']),
                        'red'))
            continue

        found = False
        for case_path in cases_path_lst:
            if label_info['ID'] in str(case_path):
```

```
                found = True
                label_info['PATH'] = case_path
                valid_lst.append(label_info)
                break
        if not found:
            print(
                colored(
                    'case {}\'s corresponding folder not found'.format(
                        label_info['ID']), 'red'))

    assert len(valid_lst) > 0
    return valid_lst
```

[12]:
```
def process_all_cases() -> None:
    """
    Tested 10.26

    Process all patient data from the dataset into *.npy files
    (Data volume: benign 183; malignant 276; total 459)

    :return: None
    """
    benign_pd = pd.concat(
        [
            pd.read_excel(
                args.excel_path / 'SK_1 -psp.xlsx')[['  ', ' ', ' mm']],
            pd.read_excel(
                args.excel_path / 'SK_1 -   .xlsx')[['  ', ' ', ' mm']],
            pd.read_excel(
                args.excel_path / 'SK_1 -   .xlsx')[['  ', ' ', ' mm']],
        ],
        axis=0,
        sort=False,
    )
    benign_label = get_label_from_pd(cases_pd=benign_pd, c=0)

    malignant_pd = pd.read_excel(args.excel_path /
                                 'SK_2 - .xlsx')[['  ', ' ', ' mm']]
    malignant_label = get_label_from_pd(cases_pd=malignant_pd, c=1)

    benign_case_path_label_list = get_filtted_case_path_and_label(
        ct_root_path=args.benign_ct_path,
        label_info_lst=benign_label,
    )
    malignant_case_path_label_list = get_filtted_case_path_and_label(
        ct_root_path=args.malignant_ct_path,
        label_info_lst=malignant_label,
```

```
    )

    if not args.output_path.exists():
        args.output_path.mkdir(parents=True, exist_ok=True)
    partial_worker = partial(
        worker_for_one_case,
        processed_path=args.output_path,
        category_names=['benign', 'malignant'],
        fixed_half_edge=args.fixed_half_edge,
        spacing=args.spacing,
    )

    print('Start processing raw dataset in parallel.')
    p = Pool()
    p.map(
        partial_worker,
        benign_case_path_label_list + malignant_case_path_label_list,
    )
    print('Finish.')
```

3.1 Extract 3D Nodule Patches from All Cases

[13]:
```
if args.pre_processing:
    process_all_cases()
```

3.2 Plot and Check Samples in Test DataSet

[14]:
```
# plt.figure(figsize=(15, 15), dpi=args.dpi)
# plt.subplots_adjust(wspace=0.35, hspace=0.35)

# count = 1
# for npy_file in (args.output_path / 'test').glob('**/*.npy'):
#     file_name, c = npy_file.stem, npy_file.parent.stem

#     x = np.load(str(npy_file)).astype(np.int16)
#     x = np.clip(x, a_min=args.wl - args.ww / 2, a_max=args.wl + args.ww / 2)
#     x = (x - (args.wl - args.ww / 2)) / args.ww
#     x = x[61:64, :, :]
#     x = x.transpose(1, 2, 0)

#     plt.subplot(7, 7, count)
#     plt.imshow(x)
#     plt.title(file_name[:-4] + '/' + str(c), fontsize=10)
#     count += 1
```

4 Build Model

4.1 Define CaseImage (Customized Class Represented for CT Case Data)

[15]:
```
def random_rotation(volume, rotation, length):
    """
    3D Affine Transform
    """
    assert isinstance(volume, np.ndarray) and volume.ndim == 3
    assert isinstance(rotation, int) and rotation > 0

    theta_x = np.pi / 180 * np.random.uniform(-rotation, rotation)
    theta_y = np.pi / 180 * np.random.uniform(-rotation, rotation)
    theta_z = np.pi / 180 * np.random.uniform(-rotation, rotation)

    rotation_matrix_x = np.array([
        [1, 0, 0],
        [0, np.cos(theta_x), -np.sin(theta_x)],
        [0, np.sin(theta_x), np.cos(theta_x)],
    ])
    rotation_matrix_y = np.array([
        [np.cos(theta_y), 0, np.sin(theta_y)],
        [0, 1, 0],
        [-np.sin(theta_y), 0, np.cos(theta_y)],
    ])
    rotation_matrix_z = np.array([
        [np.cos(theta_z), -np.sin(theta_z), 0],
        [np.sin(theta_z), np.cos(theta_z), 0],
        [0, 0, 1],
    ])
    transform_matrix = rotation_matrix_x @ rotation_matrix_y @ rotation_matrix_z

    center_in = 0.5 * np.array(volume.shape)
    center_out = 0.5 * np.array(volume.shape)

    if cp.cuda.is_available():  #affine transform on GPU
        volume_rotated = cu_ndimage.affine_transform(
            input=cp.asarray(volume),
            matrix=cp.asarray(transform_matrix),
            offset=cp.asarray(center_in - center_out.dot(transform_matrix.T)),
            order=1,
            mode='constant',
            cval=0,  #HU Value for Padding
        )
        volume_rotated = volume_rotated.get()
    else:  #affine transform on CPU
        volume_rotated = affine_transform(
            volume,
            transform_matrix,
```

```
            mode='constant',
            cval=0,  #HU Value for Padding
            offset=center_in - center_out.dot(transform_matrix.T),
            order=3,
        )
    assert isinstance(volume_rotated,
                      np.ndarray) and volume_rotated.dtype == np.int16
    return volume_rotated
```

[16]:
```
class CaseImage(fastuple):
    """
    Tested 9.11
    """
    def show(self, ctx=None, **kwargs):
        img, target = self
        assert isinstance(img, (PIL.Image.Image, Tensor))
        assert target is None or isinstance(
            target, (str, int)), f'target data type {type(target)}'

        if not isinstance(img, Tensor):
            img = tensor(img).permute(2, 0, 1)

        img = img.byte()

        line = img.new_zeros(img.shape[1], 5)
        return show_image(torch.cat([img[0], line, img[1], line, img[2]],
                                    dim=1),
                          cmap="gray",
                          figsize=(5, 15),
                          title=target,
                          ctx=ctx,
                          **kwargs)
```

4.2 Define CaseTransform (Customized Transform for Loading CT Case Data)

[17]:
```
class CaseTransform(Transform):
    """
    Tested 9.11
    """
    def __init__(self, files, label_func, splits):
        assert isinstance(files, L)
        assert hasattr(label_func, '__call__')
        assert isinstance(splits, tuple)

        self.vocab, self.label2idx = uniqueify(list(map(label_func, files)),
                                               sort=True,
                                               bidir=True)
```

```
        self.label_func = label_func
        self.train_lst = [f for f in files[splits[0]]]

    def encodes(self, fn: Path) -> CaseImage:
        assert isinstance(fn, Path) and fn.is_file(), f'Got {type(fn)}'

        f = np.load(str(fn)).astype(np.int16)

        _, h, w = f.shape
        i_middle = f.shape[0] // 2

        #step2 - (optional) affine transform
        if fn in self.train_lst:
            f = random_rotation(volume=f, rotation=180, length=h)

        #step3 - extract three slices in the middle of array
        f = np.stack(
            [
                f[i_middle, :, :],
                f[:, i_middle, :],
                f[:, :, i_middle],
            ],
            axis=0,
        )
        assert f.shape == (args.n_in, h, w), f'error in shape {f.shape}'

        #step4 - map to [0,255] and cast to uint8
        f = np.uint8(
            np.clip(
                (f - (args.wl - args.ww / 2)) / args.ww * 255,
                a_min=0,
                a_max=255,
            )).transpose((1, 2, 0))
        assert f.dtype == np.uint8

        cls = self.label2idx[self.label_func(fn)]
        return CaseImage(PILImage.create(f), cls)

    def decodes(self, x: PILImage) -> matplotlib.axes.SubplotBase:
        assert isinstance(x, PILImage), f'Wrong data type: {type(x)}'

        if not isinstance(x, Tensor):
            img = tensor(x).permute(2, 0, 1)

        img = img.byte()
        line = img.new_zeros(img.shape[1], 5)
        return show_image(
```

```
            torch.cat([img[0], line, img[1], line, img[2]], dim=1),
            cmap="gray",
            figsize=(5, 15),
        )
```

4.3 Customized Dataloaders

[18]:
```
@typedispatch
def show_batch(x: CaseImage,
               y,
               samples,
               ctxs=None,
               max_n=6,
               nrows=None,
               ncols=2,
               figsize=None,
               **kwargs):
    assert isinstance(x, CaseImage)
    assert y is None
    assert ctxs is None
    assert samples is None
    assert isinstance(max_n, int) and max_n > 0
    assert nrows is None or isinstance(nrows, int) and nrows > 0
    assert isinstance(ncols, int) and ncols > 0
    assert figsize is None or isinstance(figsize, tuple)

    if figsize is None:
        figsize = (ncols * 3, max_n // ncols * 3)
    if ctxs is None:
        ctxs = get_grid(min(x[0].shape[0], max_n),
                        nrows=None,
                        ncols=ncols,
                        figsize=figsize)
    for i, ctx in enumerate(ctxs):
        CaseImage(x[0][i], x[1][i].item()).show(ctx=ctx)


def get_dls(
    path: Path,
    train_name: str,
    valid_name: str,
) -> DataLoaders:
    assert isinstance(path, Path) and path.is_dir()
    assert isinstance(train_name, str)
    assert isinstance(valid_name, str)

    files = get_files(
```

```
        path=path,
        extensions='.npy',
        recurse=True,
    )
    splits = GrandparentSplitter(
        train_name=train_name,
        valid_name=valid_name,
    )(files)
    tfm = CaseTransform(
        files=files,
        label_func=parent_label,
        splits=splits,
    )
    tls = TfmdLists(
        files,
        tfms=[tfm],
        splits=splits,
        verbose=True,
    )

    print('Building Dataloaders')
    dataloaders = tls.dataloaders(
        after_item=[
            RandomCrop(size=args.radius_middle * 2),
            Resize(args.resize_size),
            ToTensor,
        ],
        after_batch=[
            IntToFloatTensor,
            Normalize.from_stats(*imagenet_stats),
        ],
        verbose=True,
        bs=args.batch_size,
        num_workers=args.num_workers,
    )

    return dataloaders
```

4.4 Build Training and Validation Dataset

[19]:
```
dls = get_dls(path=args.output_path, train_name='train', valid_name='valid')
test_files = get_files(
    path=args.output_path / 'test',
    extensions='.npy',
    recurse=True,
)
dl_test = dls.test_dl(test_files, shuffle=False)
```

```
Setting up Pipeline: CaseTransform
Building Dataloaders
Setting up after_item: Pipeline: RandomCrop -- {'size': (112, 112), 'p': 1.0} ->
Resize -- {'size': (224, 224), 'method': 'crop', 'pad_mode': 'reflection',
'resamples': (2, 0), 'p': 1.0} -> ToTensor
Setting up before_batch: Pipeline:
Setting up after_batch: Pipeline: IntToFloatTensor -- {'div': 255.0, 'div_mask':
1} -> Normalize -- {'mean': tensor([[[[0.4850]],

         [[0.4560]],

         [[0.4060]]]], device='cuda:0'), 'std': tensor([[[[0.2290]],

         [[0.2240]],

         [[0.2250]]]], device='cuda:0'), 'axes': (0, 2, 3)}
```

4.5 Performance Metrics

[20]:
```python
def sensitivity(y_pred, y_true, thresh=0.5):
    """
    recognized as positive if >=0.5
    """
    y_pred = F.softmax(y_pred, dim=1)[:, 1]
    TP = ((y_pred > thresh) * (y_true.data)).float().sum()
    FN = ((y_pred <= thresh) * (y_true.data)).float().sum()
    return (TP / (TP + FN)).item()


def specificity(y_pred, y_true, thresh=0.5):
    """
    recognized as positive if >=0.5
    """
    y_pred = F.softmax(y_pred, dim=1)[:, 1]
    FP = ((y_pred > thresh).float() * (1 - y_true.data).float()).sum()
    TN = ((y_pred <= thresh).float() * (1 - y_true.data).float()).sum()
    return (TN / (FP + TN)).item()


def Precision(axis=-1,
              labels=None,
              pos_label=1,
              average='binary',
              sample_weight=None):
    "Precision for single-label classification problems"
    return skm_to_fastai(
        skm.precision_score,
```

```
        axis=axis,
        labels=labels,
        pos_label=pos_label,
        average=average,
        sample_weight=sample_weight,
        zero_division=0,
    )


def Recall(axis=-1,
           labels=None,
           pos_label=1,
           average='binary',
           sample_weight=None):
    "Recall for single-label classification problems"
    return skm_to_fastai(
        skm.recall_score,
        axis=axis,
        labels=labels,
        pos_label=pos_label,
        average=average,
        sample_weight=sample_weight,
        zero_division=0,
    )
```

4.6 Build Neural Network

[21]:
```
class MyNN(Module):
    def __init__(self, encoder, head):
        self.encoder, self.head = encoder, head

    def forward(self, x):
        ftrs = self.encoder(x)
        # print(f"ftrs = {ftrs.shape}")
        cls_ = self.head(ftrs)
        # print(f"cls_ = {cls_.shape}")
        return cls_
```

[22]:
```
encoder = create_body(
    arch=resnet101,
    n_in=args.n_in,
    pretrained=True,
    cut=-2,
)
```

[23]:
```
head = create_head(512 * 4 * 1, n_out=2, ps=args.ps)
```

[24]:
```python
model = MyNN(encoder, head)


def my_nn_splitter(model):
    return [params(model.encoder), params(model.head)]
```

4.7 Loss Function

[25]:
```python
loss_func = CrossEntropyLossFlat(weight=torch.tensor(args.loss_weight).cuda())
```

4.8 Optimizer

[26]:
```python
opt_func = partial(Adam, mom=args.mom, wd=args.wd, decouple_wd=True)
```

4.9 Learner

[27]:
```python
learn = Learner(
    dls=dls,
    model=model,
    loss_func=loss_func,
    opt_func=opt_func,
    splitter=my_nn_splitter,
    path=Path('.'),
    model_dir=Path('model_weights'),
    metrics=[
        accuracy,
        RocAucBinary(),
        Recall(),
        Precision(),
    ],
    wd_bn_bias=True,
    cbs=[MixUp()],
)
learn.summary()
```

```
<IPython.core.display.HTML object>
```

[27]:
```
MyNN (Input shape: 16 x 3 x 224 x 224)
============================================================================
Layer (type)         Output Shape         Param #    Trainable
============================================================================
                     16 x 64 x 112 x 112
Conv2d                                    9408       True
BatchNorm2d                               128        True
ReLU
____________________________________________________________________________
                     16 x 64 x 56 x 56
MaxPool2d
```

```
Conv2d                                    4096       True
BatchNorm2d                               128        True
Conv2d                                    36864      True
BatchNorm2d                               128        True
____________________________________________________________________________
                     16 x 256 x 56 x 56
Conv2d                                    16384      True
BatchNorm2d                               512        True
ReLU
Conv2d                                    16384      True
BatchNorm2d                               512        True
____________________________________________________________________________
                     16 x 64 x 56 x 56
Conv2d                                    16384      True
BatchNorm2d                               128        True
Conv2d                                    36864      True
BatchNorm2d                               128        True
____________________________________________________________________________
                     16 x 256 x 56 x 56
Conv2d                                    16384      True
BatchNorm2d                               512        True
ReLU
____________________________________________________________________________
                     16 x 64 x 56 x 56
Conv2d                                    16384      True
BatchNorm2d                               128        True
Conv2d                                    36864      True
BatchNorm2d                               128        True
____________________________________________________________________________
                     16 x 256 x 56 x 56
Conv2d                                    16384      True
BatchNorm2d                               512        True
ReLU
____________________________________________________________________________
                     16 x 128 x 56 x 56
Conv2d                                    32768      True
BatchNorm2d                               256        True
____________________________________________________________________________
                     16 x 128 x 28 x 28
Conv2d                                    147456     True
BatchNorm2d                               256        True
____________________________________________________________________________
                     16 x 512 x 28 x 28
Conv2d                                    65536      True
BatchNorm2d                               1024       True
ReLU
Conv2d                                    131072     True
```

```
BatchNorm2d                                    1024        True
_____________________________________________________________________________
                    16 x 128 x 28 x 28
Conv2d                                         65536       True
BatchNorm2d                                    256         True
Conv2d                                         147456      True
BatchNorm2d                                    256         True
_____________________________________________________________________________
                    16 x 512 x 28 x 28
Conv2d                                         65536       True
BatchNorm2d                                    1024        True
ReLU
_____________________________________________________________________________
                    16 x 128 x 28 x 28
Conv2d                                         65536       True
BatchNorm2d                                    256         True
Conv2d                                         147456      True
BatchNorm2d                                    256         True
_____________________________________________________________________________
                    16 x 512 x 28 x 28
Conv2d                                         65536       True
BatchNorm2d                                    1024        True
ReLU
_____________________________________________________________________________
                    16 x 128 x 28 x 28
Conv2d                                         65536       True
BatchNorm2d                                    256         True
Conv2d                                         147456      True
BatchNorm2d                                    256         True
_____________________________________________________________________________
                    16 x 512 x 28 x 28
Conv2d                                         65536       True
BatchNorm2d                                    1024        True
ReLU
_____________________________________________________________________________
                    16 x 256 x 28 x 28
Conv2d                                         131072      True
BatchNorm2d                                    512         True
_____________________________________________________________________________
                    16 x 256 x 14 x 14
Conv2d                                         589824      True
BatchNorm2d                                    512         True
_____________________________________________________________________________
                    16 x 1024 x 14 x 14
Conv2d                                         262144      True
BatchNorm2d                                    2048        True
ReLU
```

```
Conv2d                                   524288     True
BatchNorm2d                              2048       True
____________________________________________________________________________
                     16 x 256 x 14 x 14
Conv2d                                   262144     True
BatchNorm2d                              512        True
Conv2d                                   589824     True
BatchNorm2d                              512        True
____________________________________________________________________________
                     16 x 1024 x 14 x 14
Conv2d                                   262144     True
BatchNorm2d                              2048       True
ReLU
____________________________________________________________________________
                     16 x 256 x 14 x 14
Conv2d                                   262144     True
BatchNorm2d                              512        True
Conv2d                                   589824     True
BatchNorm2d                              512        True
____________________________________________________________________________
                     16 x 1024 x 14 x 14
Conv2d                                   262144     True
BatchNorm2d                              2048       True
ReLU
____________________________________________________________________________
                     16 x 256 x 14 x 14
Conv2d                                   262144     True
BatchNorm2d                              512        True
Conv2d                                   589824     True
BatchNorm2d                              512        True
____________________________________________________________________________
                     16 x 1024 x 14 x 14
Conv2d                                   262144     True
BatchNorm2d                              2048       True
ReLU
____________________________________________________________________________
                     16 x 256 x 14 x 14
Conv2d                                   262144     True
BatchNorm2d                              512        True
Conv2d                                   589824     True
BatchNorm2d                              512        True
____________________________________________________________________________
                     16 x 1024 x 14 x 14
Conv2d                                   262144     True
BatchNorm2d                              2048       True
ReLU
____________________________________________________________________________
```

```
                     16 x 256 x 14 x 14
Conv2d                                    262144     True
BatchNorm2d                               512        True
Conv2d                                    589824     True
BatchNorm2d                               512        True
____________________________________________________________________________
                     16 x 1024 x 14 x 14
Conv2d                                    262144     True
BatchNorm2d                               2048       True
ReLU
____________________________________________________________________________
                     16 x 256 x 14 x 14
Conv2d                                    262144     True
BatchNorm2d                               512        True
Conv2d                                    589824     True
BatchNorm2d                               512        True
____________________________________________________________________________
                     16 x 1024 x 14 x 14
Conv2d                                    262144     True
BatchNorm2d                               2048       True
ReLU
____________________________________________________________________________
                     16 x 256 x 14 x 14
Conv2d                                    262144     True
BatchNorm2d                               512        True
Conv2d                                    589824     True
BatchNorm2d                               512        True
____________________________________________________________________________
                     16 x 1024 x 14 x 14
Conv2d                                    262144     True
BatchNorm2d                               2048       True
ReLU
____________________________________________________________________________
                     16 x 256 x 14 x 14
Conv2d                                    262144     True
BatchNorm2d                               512        True
Conv2d                                    589824     True
BatchNorm2d                               512        True
____________________________________________________________________________
                     16 x 1024 x 14 x 14
Conv2d                                    262144     True
BatchNorm2d                               2048       True
ReLU
____________________________________________________________________________
                     16 x 256 x 14 x 14
Conv2d                                    262144     True
BatchNorm2d                               512        True
```

```
Conv2d                                     589824     True
BatchNorm2d                                512        True

____________________________________________________________________________
                     16 x 1024 x 14 x 14
Conv2d                                     262144     True
BatchNorm2d                                2048       True
ReLU

____________________________________________________________________________
                     16 x 256 x 14 x 14
Conv2d                                     262144     True
BatchNorm2d                                512        True
Conv2d                                     589824     True
BatchNorm2d                                512        True

____________________________________________________________________________
                     16 x 1024 x 14 x 14
Conv2d                                     262144     True
BatchNorm2d                                2048       True
ReLU

____________________________________________________________________________
                     16 x 256 x 14 x 14
Conv2d                                     262144     True
BatchNorm2d                                512        True
Conv2d                                     589824     True
BatchNorm2d                                512        True

____________________________________________________________________________
                     16 x 1024 x 14 x 14
Conv2d                                     262144     True
BatchNorm2d                                2048       True
ReLU

____________________________________________________________________________
                     16 x 256 x 14 x 14
Conv2d                                     262144     True
BatchNorm2d                                512        True
Conv2d                                     589824     True
BatchNorm2d                                512        True

____________________________________________________________________________
                     16 x 1024 x 14 x 14
Conv2d                                     262144     True
BatchNorm2d                                2048       True
ReLU

____________________________________________________________________________
                     16 x 256 x 14 x 14
Conv2d                                     262144     True
BatchNorm2d                                512        True
Conv2d                                     589824     True
BatchNorm2d                                512        True

____________________________________________________________________________
```

```
                     16 x 1024 x 14 x 14
Conv2d                                    262144     True
BatchNorm2d                               2048       True
ReLU
____________________________________________________________________________
                     16 x 256 x 14 x 14
Conv2d                                    262144     True
BatchNorm2d                               512        True
Conv2d                                    589824     True
BatchNorm2d                               512        True
____________________________________________________________________________
                     16 x 1024 x 14 x 14
Conv2d                                    262144     True
BatchNorm2d                               2048       True
ReLU
____________________________________________________________________________
                     16 x 256 x 14 x 14
Conv2d                                    262144     True
BatchNorm2d                               512        True
Conv2d                                    589824     True
BatchNorm2d                               512        True
____________________________________________________________________________
                     16 x 1024 x 14 x 14
Conv2d                                    262144     True
BatchNorm2d                               2048       True
ReLU
____________________________________________________________________________
                     16 x 256 x 14 x 14
Conv2d                                    262144     True
BatchNorm2d                               512        True
Conv2d                                    589824     True
BatchNorm2d                               512        True
____________________________________________________________________________
                     16 x 1024 x 14 x 14
Conv2d                                    262144     True
BatchNorm2d                               2048       True
ReLU
____________________________________________________________________________
                     16 x 256 x 14 x 14
Conv2d                                    262144     True
BatchNorm2d                               512        True
Conv2d                                    589824     True
BatchNorm2d                               512        True
____________________________________________________________________________
                     16 x 1024 x 14 x 14
Conv2d                                    262144     True
BatchNorm2d                               2048       True
```

```
ReLU
______________________________________________________________________
                     16 x 256 x 14 x 14
Conv2d                                   262144     True
BatchNorm2d                              512        True
Conv2d                                   589824     True
BatchNorm2d                              512        True
______________________________________________________________________
                     16 x 1024 x 14 x 14
Conv2d                                   262144     True
BatchNorm2d                              2048       True
ReLU
______________________________________________________________________
                     16 x 256 x 14 x 14
Conv2d                                   262144     True
BatchNorm2d                              512        True
Conv2d                                   589824     True
BatchNorm2d                              512        True
______________________________________________________________________
                     16 x 1024 x 14 x 14
Conv2d                                   262144     True
BatchNorm2d                              2048       True
ReLU
______________________________________________________________________
                     16 x 256 x 14 x 14
Conv2d                                   262144     True
BatchNorm2d                              512        True
Conv2d                                   589824     True
BatchNorm2d                              512        True
______________________________________________________________________
                     16 x 1024 x 14 x 14
Conv2d                                   262144     True
BatchNorm2d                              2048       True
ReLU
______________________________________________________________________
                     16 x 256 x 14 x 14
Conv2d                                   262144     True
BatchNorm2d                              512        True
Conv2d                                   589824     True
BatchNorm2d                              512        True
______________________________________________________________________
                     16 x 1024 x 14 x 14
Conv2d                                   262144     True
BatchNorm2d                              2048       True
ReLU
______________________________________________________________________
                     16 x 256 x 14 x 14
```

```
Conv2d                                    262144     True
BatchNorm2d                               512        True
Conv2d                                    589824     True
BatchNorm2d                               512        True
____________________________________________________________________________
                     16 x 1024 x 14 x 14
Conv2d                                    262144     True
BatchNorm2d                               2048       True
ReLU
____________________________________________________________________________
                     16 x 512 x 14 x 14
Conv2d                                    524288     True
BatchNorm2d                               1024       True
____________________________________________________________________________
                     16 x 512 x 7 x 7
Conv2d                                    2359296    True
BatchNorm2d                               1024       True
____________________________________________________________________________
                     16 x 2048 x 7 x 7
Conv2d                                    1048576    True
BatchNorm2d                               4096       True
ReLU
Conv2d                                    2097152    True
BatchNorm2d                               4096       True
____________________________________________________________________________
                     16 x 512 x 7 x 7
Conv2d                                    1048576    True
BatchNorm2d                               1024       True
Conv2d                                    2359296    True
BatchNorm2d                               1024       True
____________________________________________________________________________
                     16 x 2048 x 7 x 7
Conv2d                                    1048576    True
BatchNorm2d                               4096       True
ReLU
____________________________________________________________________________
                     16 x 512 x 7 x 7
Conv2d                                    1048576    True
BatchNorm2d                               1024       True
Conv2d                                    2359296    True
BatchNorm2d                               1024       True
____________________________________________________________________________
                     16 x 2048 x 7 x 7
Conv2d                                    1048576    True
BatchNorm2d                               4096       True
ReLU
____________________________________________________________________________
```

```
                     16 x 2048 x 1 x 1
AdaptiveAvgPool2d
AdaptiveMaxPool2d
____________________________________________________________________________
                     16 x 4096
Flatten
BatchNorm1d                                  8192       True
Dropout
____________________________________________________________________________
                     16 x 512
Linear                                       2097152    True
ReLU
BatchNorm1d                                  1024       True
Dropout
____________________________________________________________________________
                     16 x 2
Linear                                       1024       True
____________________________________________________________________________

Total params: 44,607,552
Total trainable params: 44,607,552
Total non-trainable params: 0

Optimizer used: functools.partial(<function Adam at 0x7f560564e200>, mom=0.9,
wd=5e-05, decouple_wd=True)
Loss function: FlattenedLoss of CrossEntropyLoss()

Callbacks:
  - TrainEvalCallback
  - MixUp
  - Recorder
  - ProgressCallback
```

5 Model Training

5.1 Stage 1 Training head fine-tuning

[28]:
```python
learn.freeze()
learn.summary()
# learn.lr_find()
```

<IPython.core.display.HTML object>

[28]:
```
MyNN (Input shape: 16 x 3 x 224 x 224)
============================================================================
Layer (type)         Output Shape         Param #    Trainable
============================================================================
```

```
                     16 x 64 x 112 x 112
Conv2d                                    9408       False
BatchNorm2d                               128        True
ReLU
____________________________________________________________________________
                     16 x 64 x 56 x 56
MaxPool2d
Conv2d                                    4096       False
BatchNorm2d                               128        True
Conv2d                                    36864      False
BatchNorm2d                               128        True
____________________________________________________________________________
                     16 x 256 x 56 x 56
Conv2d                                    16384      False
BatchNorm2d                               512        True
ReLU
Conv2d                                    16384      False
BatchNorm2d                               512        True
____________________________________________________________________________
                     16 x 64 x 56 x 56
Conv2d                                    16384      False
BatchNorm2d                               128        True
Conv2d                                    36864      False
BatchNorm2d                               128        True
____________________________________________________________________________
                     16 x 256 x 56 x 56
Conv2d                                    16384      False
BatchNorm2d                               512        True
ReLU
____________________________________________________________________________
                     16 x 64 x 56 x 56
Conv2d                                    16384      False
BatchNorm2d                               128        True
Conv2d                                    36864      False
BatchNorm2d                               128        True
____________________________________________________________________________
                     16 x 256 x 56 x 56
Conv2d                                    16384      False
BatchNorm2d                               512        True
ReLU
____________________________________________________________________________
                     16 x 128 x 56 x 56
Conv2d                                    32768      False
BatchNorm2d                               256        True
____________________________________________________________________________
                     16 x 128 x 28 x 28
Conv2d                                    147456     False
```

```
BatchNorm2d                                  256        True
____________________________________________________________________________
                     16 x 512 x 28 x 28
Conv2d                                       65536      False
BatchNorm2d                                  1024       True
ReLU
Conv2d                                       131072     False
BatchNorm2d                                  1024       True
____________________________________________________________________________
                     16 x 128 x 28 x 28
Conv2d                                       65536      False
BatchNorm2d                                  256        True
Conv2d                                       147456     False
BatchNorm2d                                  256        True
____________________________________________________________________________
                     16 x 512 x 28 x 28
Conv2d                                       65536      False
BatchNorm2d                                  1024       True
ReLU
____________________________________________________________________________
                     16 x 128 x 28 x 28
Conv2d                                       65536      False
BatchNorm2d                                  256        True
Conv2d                                       147456     False
BatchNorm2d                                  256        True
____________________________________________________________________________
                     16 x 512 x 28 x 28
Conv2d                                       65536      False
BatchNorm2d                                  1024       True
ReLU
____________________________________________________________________________
                     16 x 128 x 28 x 28
Conv2d                                       65536      False
BatchNorm2d                                  256        True
Conv2d                                       147456     False
BatchNorm2d                                  256        True
____________________________________________________________________________
                     16 x 512 x 28 x 28
Conv2d                                       65536      False
BatchNorm2d                                  1024       True
ReLU
____________________________________________________________________________
                     16 x 256 x 28 x 28
Conv2d                                       131072     False
BatchNorm2d                                  512        True
____________________________________________________________________________
                     16 x 256 x 14 x 14
```

```
Conv2d                                  589824     False
BatchNorm2d                             512        True

----------------------------------------------------------------------------
                     16 x 1024 x 14 x 14
Conv2d                                  262144     False
BatchNorm2d                             2048       True
ReLU
Conv2d                                  524288     False
BatchNorm2d                             2048       True

----------------------------------------------------------------------------
                     16 x 256 x 14 x 14
Conv2d                                  262144     False
BatchNorm2d                             512        True
Conv2d                                  589824     False
BatchNorm2d                             512        True

----------------------------------------------------------------------------
                     16 x 1024 x 14 x 14
Conv2d                                  262144     False
BatchNorm2d                             2048       True
ReLU

----------------------------------------------------------------------------
                     16 x 256 x 14 x 14
Conv2d                                  262144     False
BatchNorm2d                             512        True
Conv2d                                  589824     False
BatchNorm2d                             512        True

----------------------------------------------------------------------------
                     16 x 1024 x 14 x 14
Conv2d                                  262144     False
BatchNorm2d                             2048       True
ReLU

----------------------------------------------------------------------------
                     16 x 256 x 14 x 14
Conv2d                                  262144     False
BatchNorm2d                             512        True
Conv2d                                  589824     False
BatchNorm2d                             512        True

----------------------------------------------------------------------------
                     16 x 1024 x 14 x 14
Conv2d                                  262144     False
BatchNorm2d                             2048       True
ReLU

----------------------------------------------------------------------------
                     16 x 256 x 14 x 14
Conv2d                                  262144     False
BatchNorm2d                             512        True
Conv2d                                  589824     False
```

```
BatchNorm2d                                 512        True

____________________________________________________________________________
                     16 x 1024 x 14 x 14
Conv2d                                      262144     False
BatchNorm2d                                 2048       True
ReLU

____________________________________________________________________________
                     16 x 256 x 14 x 14
Conv2d                                      262144     False
BatchNorm2d                                 512        True
Conv2d                                      589824     False
BatchNorm2d                                 512        True

____________________________________________________________________________
                     16 x 1024 x 14 x 14
Conv2d                                      262144     False
BatchNorm2d                                 2048       True
ReLU

____________________________________________________________________________
                     16 x 256 x 14 x 14
Conv2d                                      262144     False
BatchNorm2d                                 512        True
Conv2d                                      589824     False
BatchNorm2d                                 512        True

____________________________________________________________________________
                     16 x 1024 x 14 x 14
Conv2d                                      262144     False
BatchNorm2d                                 2048       True
ReLU

____________________________________________________________________________
                     16 x 256 x 14 x 14
Conv2d                                      262144     False
BatchNorm2d                                 512        True
Conv2d                                      589824     False
BatchNorm2d                                 512        True

____________________________________________________________________________
                     16 x 1024 x 14 x 14
Conv2d                                      262144     False
BatchNorm2d                                 2048       True
ReLU

____________________________________________________________________________
                     16 x 256 x 14 x 14
Conv2d                                      262144     False
BatchNorm2d                                 512        True
Conv2d                                      589824     False
BatchNorm2d                                 512        True

____________________________________________________________________________
                     16 x 1024 x 14 x 14
```

```
Conv2d                                    262144     False
BatchNorm2d                               2048       True
ReLU
____________________________________________________________________________
                     16 x 256 x 14 x 14
Conv2d                                    262144     False
BatchNorm2d                               512        True
Conv2d                                    589824     False
BatchNorm2d                               512        True
____________________________________________________________________________
                     16 x 1024 x 14 x 14
Conv2d                                    262144     False
BatchNorm2d                               2048       True
ReLU
____________________________________________________________________________
                     16 x 256 x 14 x 14
Conv2d                                    262144     False
BatchNorm2d                               512        True
Conv2d                                    589824     False
BatchNorm2d                               512        True
____________________________________________________________________________
                     16 x 1024 x 14 x 14
Conv2d                                    262144     False
BatchNorm2d                               2048       True
ReLU
____________________________________________________________________________
                     16 x 256 x 14 x 14
Conv2d                                    262144     False
BatchNorm2d                               512        True
Conv2d                                    589824     False
BatchNorm2d                               512        True
____________________________________________________________________________
                     16 x 1024 x 14 x 14
Conv2d                                    262144     False
BatchNorm2d                               2048       True
ReLU
____________________________________________________________________________
                     16 x 256 x 14 x 14
Conv2d                                    262144     False
BatchNorm2d                               512        True
Conv2d                                    589824     False
BatchNorm2d                               512        True
____________________________________________________________________________
                     16 x 1024 x 14 x 14
Conv2d                                    262144     False
BatchNorm2d                               2048       True
ReLU
```

```
----------------------------------------------------------------------------
                     16 x 256 x 14 x 14
Conv2d                                    262144     False
BatchNorm2d                               512        True
Conv2d                                    589824     False
BatchNorm2d                               512        True
----------------------------------------------------------------------------
                     16 x 1024 x 14 x 14
Conv2d                                    262144     False
BatchNorm2d                               2048       True
ReLU
----------------------------------------------------------------------------
                     16 x 256 x 14 x 14
Conv2d                                    262144     False
BatchNorm2d                               512        True
Conv2d                                    589824     False
BatchNorm2d                               512        True
----------------------------------------------------------------------------
                     16 x 1024 x 14 x 14
Conv2d                                    262144     False
BatchNorm2d                               2048       True
ReLU
----------------------------------------------------------------------------
                     16 x 256 x 14 x 14
Conv2d                                    262144     False
BatchNorm2d                               512        True
Conv2d                                    589824     False
BatchNorm2d                               512        True
----------------------------------------------------------------------------
                     16 x 1024 x 14 x 14
Conv2d                                    262144     False
BatchNorm2d                               2048       True
ReLU
----------------------------------------------------------------------------
                     16 x 256 x 14 x 14
Conv2d                                    262144     False
BatchNorm2d                               512        True
Conv2d                                    589824     False
BatchNorm2d                               512        True
----------------------------------------------------------------------------
                     16 x 1024 x 14 x 14
Conv2d                                    262144     False
BatchNorm2d                               2048       True
ReLU
----------------------------------------------------------------------------
                     16 x 256 x 14 x 14
Conv2d                                    262144     False
```

```
BatchNorm2d                                   512        True
Conv2d                                        589824     False
BatchNorm2d                                   512        True
____________________________________________________________________________
                     16 x 1024 x 14 x 14
Conv2d                                        262144     False
BatchNorm2d                                   2048       True
ReLU
____________________________________________________________________________
                     16 x 256 x 14 x 14
Conv2d                                        262144     False
BatchNorm2d                                   512        True
Conv2d                                        589824     False
BatchNorm2d                                   512        True
____________________________________________________________________________
                     16 x 1024 x 14 x 14
Conv2d                                        262144     False
BatchNorm2d                                   2048       True
ReLU
____________________________________________________________________________
                     16 x 256 x 14 x 14
Conv2d                                        262144     False
BatchNorm2d                                   512        True
Conv2d                                        589824     False
BatchNorm2d                                   512        True
____________________________________________________________________________
                     16 x 1024 x 14 x 14
Conv2d                                        262144     False
BatchNorm2d                                   2048       True
ReLU
____________________________________________________________________________
                     16 x 256 x 14 x 14
Conv2d                                        262144     False
BatchNorm2d                                   512        True
Conv2d                                        589824     False
BatchNorm2d                                   512        True
____________________________________________________________________________
                     16 x 1024 x 14 x 14
Conv2d                                        262144     False
BatchNorm2d                                   2048       True
ReLU
____________________________________________________________________________
                     16 x 256 x 14 x 14
Conv2d                                        262144     False
BatchNorm2d                                   512        True
Conv2d                                        589824     False
BatchNorm2d                                   512        True
```

```
--------------------------------------------------------------------------------
                      16 x 1024 x 14 x 14
Conv2d                                     262144     False
BatchNorm2d                                2048       True
ReLU
--------------------------------------------------------------------------------
                      16 x 256 x 14 x 14
Conv2d                                     262144     False
BatchNorm2d                                512        True
Conv2d                                     589824     False
BatchNorm2d                                512        True
--------------------------------------------------------------------------------
                      16 x 1024 x 14 x 14
Conv2d                                     262144     False
BatchNorm2d                                2048       True
ReLU
--------------------------------------------------------------------------------
                      16 x 512 x 14 x 14
Conv2d                                     524288     False
BatchNorm2d                                1024       True
--------------------------------------------------------------------------------
                      16 x 512 x 7 x 7
Conv2d                                     2359296    False
BatchNorm2d                                1024       True
--------------------------------------------------------------------------------
                      16 x 2048 x 7 x 7
Conv2d                                     1048576    False
BatchNorm2d                                4096       True
ReLU
Conv2d                                     2097152    False
BatchNorm2d                                4096       True
--------------------------------------------------------------------------------
                      16 x 512 x 7 x 7
Conv2d                                     1048576    False
BatchNorm2d                                1024       True
Conv2d                                     2359296    False
BatchNorm2d                                1024       True
--------------------------------------------------------------------------------
                      16 x 2048 x 7 x 7
Conv2d                                     1048576    False
BatchNorm2d                                4096       True
ReLU
--------------------------------------------------------------------------------
                      16 x 512 x 7 x 7
Conv2d                                     1048576    False
BatchNorm2d                                1024       True
Conv2d                                     2359296    False
```

```
BatchNorm2d                                1024       True
____________________________________________________________________________
                     16 x 2048 x 7 x 7
Conv2d                                     1048576    False
BatchNorm2d                                4096       True
ReLU
____________________________________________________________________________
                     16 x 2048 x 1 x 1
AdaptiveAvgPool2d
AdaptiveMaxPool2d
____________________________________________________________________________
                     16 x 4096
Flatten
BatchNorm1d                                8192       True
Dropout
____________________________________________________________________________
                     16 x 512
Linear                                     2097152    True
ReLU
BatchNorm1d                                1024       True
Dropout
____________________________________________________________________________
                     16 x 2
Linear                                     1024       True
____________________________________________________________________________

Total params: 44,607,552
Total trainable params: 2,212,736
Total non-trainable params: 42,394,816

Optimizer used: functools.partial(<function Adam at 0x7f560564e200>, mom=0.9,
wd=5e-05, decouple_wd=True)
Loss function: FlattenedLoss of CrossEntropyLoss()

Model frozen up to parameter group #1

Callbacks:
  - TrainEvalCallback
  - MixUp
  - Recorder
  - ProgressCallback
```

[29]:
```
learn.fine_tune(
    epochs=args.epoch,
    base_lr=args.lr,
    freeze_epochs=1,
    lr_mult=100,
```

```
    pct_start=0.3,
    div=5.0,
    div_final=100000.0,
    cbs=[
        SaveModelCallback(
            monitor='valid_loss',
            fname=f'stage1-bestmodel-{args.exp_name}',
            with_opt=False,
        )
    ],
    reset_opt=False,
)
```

```
<IPython.core.display.HTML object>

Better model found at epoch 0 with valid_loss value: 1.7147160768508911.

<IPython.core.display.HTML object>

Better model found at epoch 0 with valid_loss value: 0.6074032783508301.
Better model found at epoch 3 with valid_loss value: 0.5394479632377625.
Better model found at epoch 4 with valid_loss value: 0.5021474957466125.
Better model found at epoch 7 with valid_loss value: 0.48461371660232544.
Better model found at epoch 30 with valid_loss value: 0.4696860909461975.
Better model found at epoch 31 with valid_loss value: 0.46278977394104004.
Better model found at epoch 40 with valid_loss value: 0.44813910126686096.
Better model found at epoch 43 with valid_loss value: 0.43726667761802673.
Better model found at epoch 49 with valid_loss value: 0.4330184757709503.
Better model found at epoch 53 with valid_loss value: 0.4274168312549591.
```

6 Plot the Results on the Three Datasets

6.1 Results on Training Dataset

[30]:
```
def plot_top_losses(x,
                    y,
                    samples,
                    outs,
                    raws,
                    losses,
                    nrows=None,
                    ncols=None,
                    figsize=None,
                    **kwargs):
    axs = get_grid(len(samples),
                   nrows=nrows,
                   ncols=ncols,
                   add_vert=1,
                   figsize=figsize,
```

```
                  title='Prediction/Actual/Loss/Probability',
                  dpi=args.dpi)
    for ax, s, o, r, l in zip(axs, samples, outs, raws, losses):
        s[0].show(ctx=ax, **kwargs)
        ax.set_title(
            f'{o[0].argmax(-1)}/{s[1]} / {l.item():.2f} / {r.max().item():.2f}'
        )


class MyInterpretation(Interpretation):
    "Interpretation base class, can be inherited for task specific␣
 ↪Interpretation classes"

    def __init__(self, dl, inputs, preds, targs, decoded, losses):
        super().__init__(dl, inputs, preds, targs, decoded, losses)

    def _pre_show_batch(self, b, max_n=9):
        "Decode `b` to be ready for `show_batch`"
        b = self.dl.decode(b)
        its = L()
        for x1, y1 in zip(b[0], b[1]):
            its.append((CaseImage(x1, None), y1))
        if not is_listy(b):
            b, its = [b], L((o, ) for o in its)
        return detuplify(b[:self.dl.n_inp]), detuplify(b[self.dl.n_inp:]), its

    @classmethod
    def from_learner(cls, learn, ds_idx=1, dl=None, act=None):
        "Construct interpretation object from a learner"
        if ds_idx in (0, 1):
            return cls(
                dl,
                *learn.get_preds(ds_idx=ds_idx,
                                 with_input=True,
                                 with_loss=True,
                                 with_decoded=True,
                                 act=None))
        else:
            return cls(
                dl,
                *learn.get_preds(dl=dl,
                                 with_input=True,
                                 with_loss=True,
                                 with_decoded=True,
                                 act=None))

    def plot_top_losses(self, k, largest=True, **kwargs):
```

```python
        losses, idx = self.top_losses(k, largest)
        if not isinstance(self.inputs, tuple):
            self.inputs = (self.inputs, )

        if isinstance(self.inputs[0], Tensor):
            inps = tuple(o[idx] for o in self.inputs)
        else:
            inps = self.dl.create_batch(
                self.dl.before_batch(
                    [tuple(o[i] for o in self.inputs) for i in idx]))

        b = inps + tuple(
            o[idx]
            for o in (self.targs if is_listy(self.targs) else (self.targs, )))

        x, y, its = self._pre_show_batch(b, max_n=k)

        b_out = inps + tuple(o[idx] for o in (
            self.decoded if is_listy(self.decoded) else (self.decoded, )))

        x1, y1, outs = self._pre_show_batch(b_out, max_n=k)
        if its is not None:
            plot_top_losses(x, y, its, outs.itemgot(slice(len(inps), None)),
                            self.preds[idx], losses, **kwargs)


class MyClassificationInterpretation(MyInterpretation):
    "Interpretation methods for classification models."

    def __init__(self, dl, inputs, preds, targs, decoded, losses):
        super().__init__(dl, inputs, preds, targs, decoded, losses)
        self.vocab = self.dl.vocab

    def confusion_matrix(self):
        "Confusion matrix as an `np.ndarray`."
        x = torch.arange(0, len(self.vocab))
        d, t = flatten_check(self.decoded.argmax(-1), self.targs)
        cm = ((d == x[:, None]) & (t == x[:, None, None])).long().sum(2)
        return to_np(cm)

    def plot_confusion_matrix(self,
                              normalize=False,
                              title='Confusion matrix',
                              cmap="Blues",
                              norm_dec=2,
                              plot_txt=True,
                              **kwargs):
```

```python
        "Plot the confusion matrix, with `title` and using `cmap`."
        cm = self.confusion_matrix()
        if normalize: cm = cm.astype('float') / cm.sum(axis=1)[:, np.newaxis]
        fig = plt.figure(**kwargs)
        plt.imshow(cm, interpolation='nearest', cmap=cmap)
        plt.title(title)
        tick_marks = np.arange(len(self.vocab))
        plt.xticks(tick_marks, self.vocab, rotation=90)
        plt.yticks(tick_marks, self.vocab, rotation=0)

        if plot_txt:
            thresh = cm.max() / 2.
            for i, j in itertools.product(range(cm.shape[0]),
                                          range(cm.shape[1])):
                coeff = f'{cm[i, j]:.{norm_dec}f}' if normalize else f'{cm[i,␣
↪j]}'
                plt.text(j,
                         i,
                         coeff,
                         horizontalalignment="center",
                         verticalalignment="center",
                         color="white" if cm[i, j] > thresh else "black")

        ax = fig.gca()
        ax.set_ylim(len(self.vocab) - .5, -.5)

        plt.tight_layout()
        plt.ylabel('Actual')
        plt.xlabel('Predicted')
        plt.grid(False)

    def most_confused(self, min_val=1):
        "Sorted descending list of largest non-diagonal entries of confusion␣
↪matrix, presented as actual, predicted, number of occurrences."
        cm = self.confusion_matrix()
        np.fill_diagonal(cm, 0)
        res = [(self.vocab[i], self.vocab[j], cm[i, j])
               for i, j in zip(*np.where(cm >= min_val))]
        return sorted(res, key=itemgetter(2), reverse=True)

    def print_classification_report(self):
        "Print scikit-learn classification report"
        d, t = flatten_check(self.decoded, self.targs)
        print(
            skm.classification_report(
                t,
                d,
```

```python
                labels=list(self.vocab.o2i.values()),
                target_names=[str(v) for v in self.vocab]))


def validate_one_set(learn, dl, ds_idx=-1, dataset_name=None):
    assert isinstance(ds_idx, int) and ds_idx in (-1, 0, 1)
    assert isinstance(dataset_name, str)

    res = learn.validate(ds_idx=ds_idx, dl=dl, cbs=None)
    print(
        f'{dataset_name}: loss = {res[0]}, accuracy = {res[1]}, auc = {res[2]},␣
↪sensitivity(recall) = {res[3]}, precision = {res[4]}'
    )

    # interp = MyClassificationInterpretation.from_learner(
    #     learn=learn,
    #     dl=dl,
    #     ds_idx=ds_idx,
    # )
    # interp.plot_confusion_matrix(
    #     title=f'Confusion Matrix of {dataset_name} Set', dpi=args.dpi)

    # interp.plot_top_losses(k=25, largest=True, figsize=(30, 10))


class Hook():
    """
    Tested 9.12
    """
    def __init__(self, m):
        self.hook = m.register_forward_hook(self.hook_func)

    def hook_func(self, m, i, o):
        self.stored = o.detach().clone()

    def __enter__(self, *args):
        return self

    def __exit__(self, *args):
        self.hook.remove()


class HookBwd():
    """
    Tested 9.12
    """
    def __init__(self, m):
```

```python
        self.hook = m.register_backward_hook(self.hook_func)

    def hook_func(self, m, gi, go):
        self.stored = go[0].detach().clone()

    def __enter__(self, *args):
        return self

    def __exit__(self, *args):
        self.hook.remove()


def interpret(dl, cls, x_idx, layer_idx, ax):
    """
    Tested 9.12
    """

    xs, ys = first(dl)
    x = xs[x_idx][None]
    print('target: ', ys[x_idx])
    x_dec = dl.decode((x, ))[0][0]
    with HookBwd(learn.model.encoder[layer_idx]) as hookg:
        with Hook(learn.model.encoder[layer_idx]) as hook:
            output = learn.model.eval()(x.cuda())
            print('prediction: ', F.softmax(output, dim=-1))
            act = hook.stored
        output[0, cls].backward()
        grad = hookg.stored

    w = grad[0].mean(dim=[1, 2], keepdim=True)
    cam_map = (w * act[0]).sum(0)
    CaseImage(x_dec, None).show(ctx=ax)

    line = x_dec.new_zeros(x_dec.shape[1], 5)
    cam_map = cam_map.detach().cpu()
    cam_map = tensor(
        cv2.resize(cam_map.numpy(),
                   dsize=x_dec.shape[-2:],
                   interpolation=cv2.INTER_CUBIC))
    im = ax.imshow(
        torch.cat([cam_map, line, cam_map, line, cam_map], dim=1),
        alpha=0.5,
        cmap='jet',
        vmin=-0.05,
        vmax=0.05,
    )
```

```python
    return im


def interpret_one(dl, layer_idx, ids, title):
    """
    Tested 9.12
    """
    fig, axs = plt.subplots(nrows=3, ncols=1, figsize=(30, 10), dpi=args.dpi)
    for i, ax in enumerate(axs.reshape(-1)):
        if i == 0:
            xs, ys = first(dl)
            x = xs[ids[0]][None]  #get x in the first case
            x_dec = dl.decode((x, ))[0][0]
            CaseImage(x_dec, None).show(ctx=ax)
        elif i == 1:
            im = interpret(dl=dl,
                           cls=0,
                           x_idx=ids[0],
                           layer_idx=layer_idx,
                           ax=ax)
        elif i == 2:
            im = interpret(dl=dl,
                           cls=1,
                           x_idx=ids[0],
                           layer_idx=layer_idx,
                           ax=ax)

    cbar_ax = fig.add_axes([0.65, 0.15, 0.025, 0.7])
    cbar = fig.colorbar(
        im,
        cax=cbar_ax,
    )

    cbar.ax.tick_params(labelsize=0, axis='both', which='both', length=0)

    plt.show()
```

[31]:
```python
if args.plot_validate_result:
    # learn.load("stage1-bestmodel-SK1028")
    validate_one_set(
        learn=learn,
        dl=dls.train,
        ds_idx=0,
        dataset_name='Train',
    )
```

```
<IPython.core.display.HTML object>
```

Train: loss = 0.2938244342803955, accuracy = 0.90625, auc = 0.9484581349005302, sensitivity(recall) = 0.9389671361502347, precision = 0.9090909090909091

6.2 Results on Validation Dataset

[32]:
```
if args.plot_validate_result:
    validate_one_set(
        learn=learn,
        dl=dls.valid,
        dataset_name='Validation',
    )
```

<IPython.core.display.HTML object>

Validation: loss = 0.4274168312549591, accuracy = 0.8695651888847351, auc = 0.861111111111111, sensitivity(recall) = 0.9285714285714286, precision = 0.8666666666666667

6.3 Results on Test Dataset

[33]:
```
if args.plot_validate_result:
    # learn.load("stage1-bestmodel-SK1028")
    validate_one_set(
        learn=learn,
        dl=dl_test,
        dataset_name='Test',
    )
    # ids = [14]
    # interpret_one(dl=dl_test, layer_idx=-1, ids=ids, title='Test')
    # random.seed(8)
    # ids = random.sample(range(min(args.batch_size, len(dl_test.items))), 1)
    # print(f"ids = {ids}")
    # interpret_one(dl=dl_test, layer_idx=-1, ids=ids, title='Test')
    # random.seed(24)  #12
    # ids = random.sample(range(min(args.batch_size, len(dl_test.items))), 1)
    # interpret_one(dl=dl_test, layer_idx=-1, ids=ids, title='Test')
```

<IPython.core.display.HTML object>

Test: loss = 0.3608114421367645, accuracy = 0.8297872543334961, auc = 0.9097744360902256, sensitivity(recall) = 0.8928571428571429, precision = 0.8333333333333334

7 Others

7.1 Plot Histogram

[34]:
```
# def plot_histogram(path: Path, bins: int = 30) -> None:
#     assert isinstance(path, Path) and path.is_dir()
#     assert isinstance(bins, int) and bins > 0

#     train_path = path / 'train'
#     valid_path = path / 'valid'
#     test_path = path / 'test'

#     assert isinstance(train_path, Path) and train_path.is_dir()
#     assert isinstance(valid_path, Path) and valid_path.is_dir()
#     assert isinstance(test_path, Path) and test_path.is_dir()

#     benign_pd = pd.concat(
#         [
#             pd.read_excel(
#                 args.excel_path / 'SK_1-psp.xlsx')[['  ', ' ', ' mm']],
#             pd.read_excel(
#                 args.excel_path / 'SK_1-   .xlsx')[['  ', ' ', ' mm']],
#             pd.read_excel(
#                 args.excel_path / 'SK_1-   .xlsx')[['  ', ' ', ' mm']],
#         ],
#         axis=0,
#         sort=False,
#     )
#     benign_label = get_label_from_pd(cases_pd=benign_pd, c=0)
#     malignant_pd = pd.read_excel(args.excel_path /
#                                  'SK_2- .xlsx')[['  ', ' ', ' mm']]
#     malignant_label = get_label_from_pd(cases_pd=malignant_pd, c=1)
#     case_path = [
#         p for p in args.malignant_ct_path.glob('*/*') if p.is_dir()
#     ] + [p for p in args.benign_ct_path.glob('*/*') if p.is_dir()]
#     all_label = benign_label + malignant_label

#     ids = {
#         'train': {
#             'benign': [],
#             'malignant': [],
#         },
#         'valid': {
#             'benign': [],
#             'malignant': [],
#         },
#         'test': {
#             'benign': [],
#             'malignant': [],
```

```
#         }
#     }

#     for sub_set_path in path.ls():
#         for category_path in sub_set_path.ls():
#             for one_case_path in category_path.ls():
#                 sub_set_name = one_case_path.parent.parent.stem
#                 category_name = one_case_path.parent.stem
#                 case_name = one_case_path.stem

#                 indices = [
#                     i for i, s in enumerate(case_path) if case_name in str(s)
#                 ]
#                 assert len(indices) == 1

#                 one_slice = sitk.ReadImage(
#                     str(list(case_path[indices[0]].glob('*.dcm'))[0]))
#                 spacing = np.array(list(reversed(one_slice.GetSpacing())))
#                 indices = [
#                     i for i, d in enumerate(all_label) if case_name in d['ID']
#                 ]
#                 assert len(indices) == 1

#                 new_r = all_label[
#                     indices[0]]['R'] / spacing[2] * args.spacing[2]
#                 ids[sub_set_name][category_name].append(new_r)

#             plt.figure(figsize=(4, 4), dpi=args.dpi)
#             plt.hist(
#                 ids[str(sub_set_path.stem)][str(category_path.stem)],
#                 bins=bins,
#                 facecolor="blue",
#                 edgecolor="black",
#                 alpha=0.7,
#                 density=True,
#                 range=[0, bins],
#             )
#             plt.xlabel("r (in pixels)")
#             plt.ylabel("freq")
#             plt.title(f'{str(sub_set_path.stem)}/{str(category_path.stem)}')
#             plt.show()


# plot_histogram(args.output_path)
```

7.2 Save Predicts to Excel

[35]:
```
# def save_prediction_excel(ids, pred, targs, excel_path):
#     """
#     Output prediction results of train, valid and test dataset to a excel␣
↪file.
#     """
#     df = pd.DataFrame(
#         np.concatenate((ids[:, np.newaxis], pred, targs[:, np.newaxis]),
#                        axis=-1))
#     df.to_excel(excel_path, index=False)
```

[36]:
```
# #train set
# preds, targets = learn.get_preds(ds_idx=0, reorder=True)
# ids = np.array([str(i).split('/')[-1].split('.')[0] for i in dls.train.items])

# save_prediction_excel(
#     ids,
#     F.softmax(preds, dim=-1).cpu().numpy(),
#     targets,
#     args.excel_path / 'predict_train.xlsx',
# )

# #validation set
# preds, targets = learn.get_preds(dl=dls.valid)
# ids = np.array([str(i).split('/')[-1].split('.')[0] for i in dls.valid.items])

# save_prediction_excel(
#     ids,
#     F.softmax(preds, dim=-1).cpu().numpy(),
#     targets,
#     args.excel_path / 'predict_valid.xlsx',
# )

# #test set
# preds, targets = learn.get_preds(dl=dl_test)
# ids = np.array([str(i).split('/')[-1].split('.')[0] for i in dl_test.items])

# save_prediction_excel(
#     ids,
#     F.softmax(preds, dim=-1).cpu().numpy(),
#     targets,
#     args.excel_path / 'predict_test.xlsx',
# )
```

7.3 Plot ROC Curve

[37]:
```
fpr = dict()
tpr = dict()
roc_auc = dict()

# For Test Set
preds, targets = learn.get_preds(dl=dl_test)
fpr[0], tpr[0], _ = skm.roc_curve(
    y_true=targets,
    y_score=F.softmax(preds, dim=-1).cpu().numpy()[:, 1],
)
roc_auc[0] = skm.auc(fpr[0], tpr[0])

#For Valid Set
preds, targets = learn.get_preds(dl=dls.valid)
fpr[1], tpr[1], _ = skm.roc_curve(
    y_true=targets,
    y_score=F.softmax(preds, dim=-1).cpu().numpy()[:, 1],
)
roc_auc[1] = skm.auc(fpr[1], tpr[1])

#For Train Set
preds, targets = learn.get_preds(ds_idx=0, reorder=True)
fpr[2], tpr[2], _ = skm.roc_curve(
    y_true=targets,
    y_score=F.softmax(preds, dim=-1).cpu().numpy()[:, 1],
)
roc_auc[2] = skm.auc(fpr[2], tpr[2])

plt.figure(dpi=args.dpi, figsize=(5, 5))
lw = 2
plt.plot(fpr[2],
         tpr[2],
         color='blue',
         lw=lw,
         label='Train Set ROC curve (AUC = %0.2f)' % roc_auc[2])
plt.plot(fpr[1],
         tpr[1],
         color='green',
         lw=lw,
         label='Validation Set ROC curve (AUC = %0.2f)' % roc_auc[1])
plt.plot(fpr[0],
         tpr[0],
         color='darkorange',
         lw=lw,
         label='Test Set ROC curve (AUC = %0.2f)' % roc_auc[0])
```

```python
plt.plot([0, 1], [0, 1], color='navy', lw=lw, linestyle='--')
plt.xlim([0.0, 1.0])
plt.ylim([0.0, 1.05])
plt.xlabel(
    '1 - Specificity',
    fontsize=11,
)
plt.ylabel(
    'Sensitivity',
    fontsize=11,
)
plt.title(
    'Receiver Operating Characteristic',
    fontsize=11,
)
plt.legend(loc="lower right")
plt.show()
```

```
<IPython.core.display.HTML object>

<IPython.core.display.HTML object>

<IPython.core.display.HTML object>

findfont: Font family ['Times New Roman'] not found. Falling back to DejaVu
Sans.
findfont: Font family ['Times New Roman'] not found. Falling back to DejaVu
Sans.
```

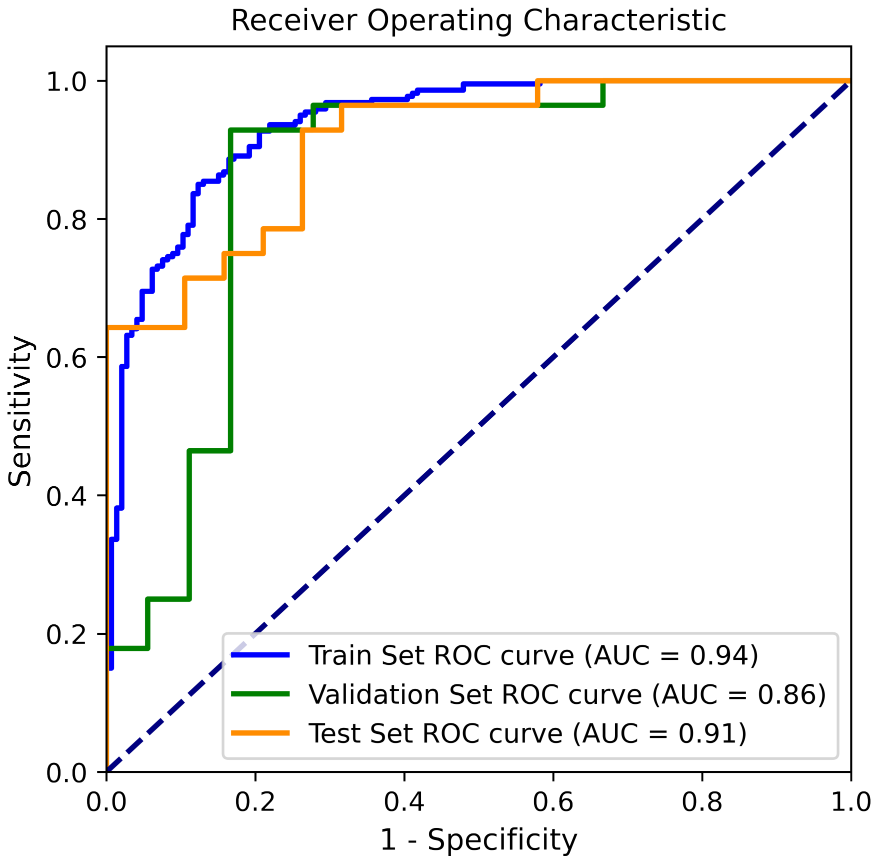

Receiver Operating Characteristic
Sensitivity
1 - Specificity
0.0
0.2
0.4
0.6
0.8
1.0
Train Set ROC curve (AUC = 0.94)
Validation Set ROC curve (AUC = 0.86)
Test Set ROC curve (AUC = 0.91)

[ ]:
