# Supplementary figures and images for: Convolutional Neural Network-Based Diagnostic Model for a Solid, Indeterminate Solitary Pulmonary Nodule or Mass on Computed Tomography
Source: Front Oncol. 2021 Dec 21;11:792062. doi: 10.3389/fonc.2021.792062 (PMC8724915; doi:10.3389/fonc.2021.792062)

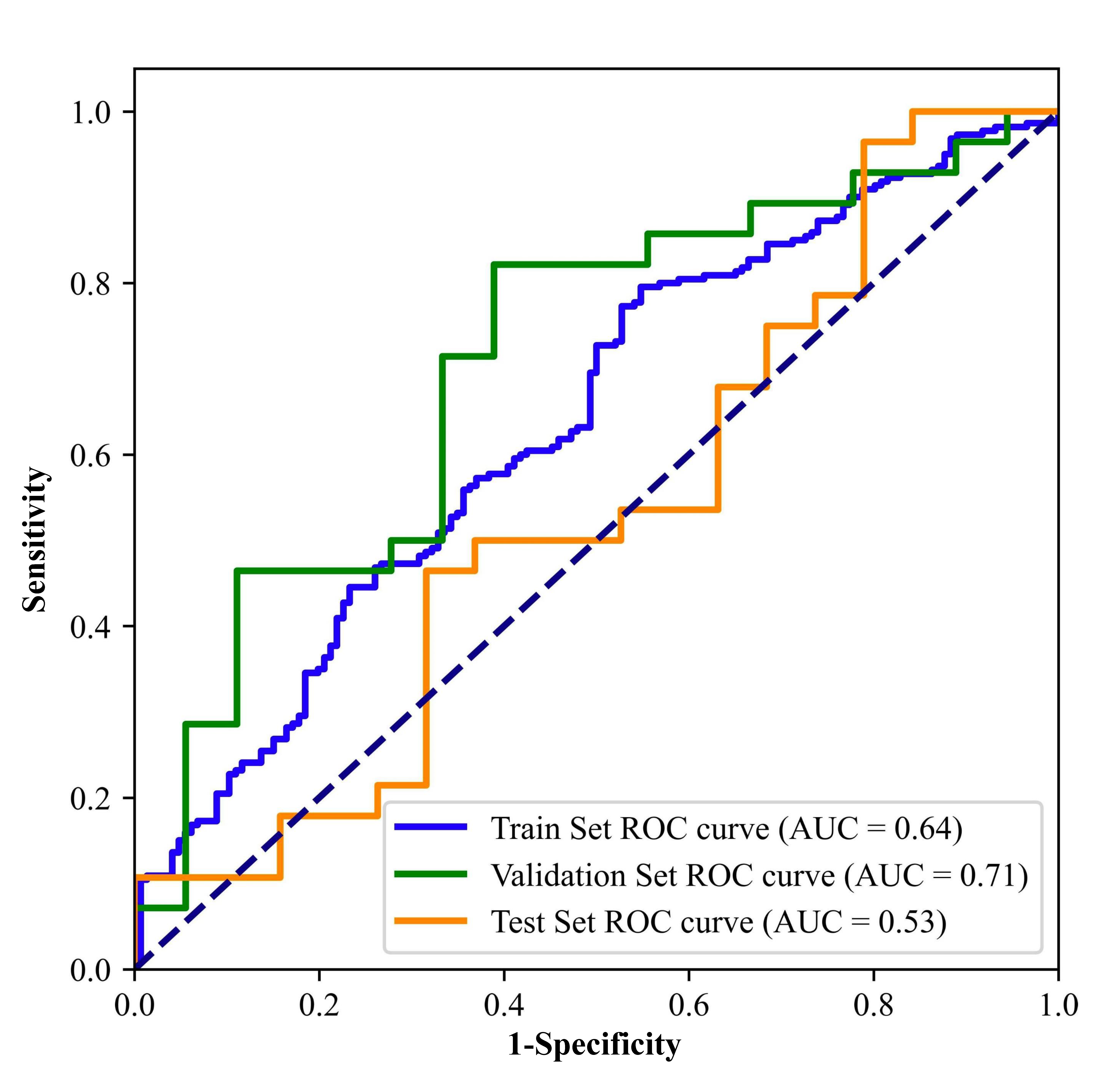

Supplement: Supplementary Figure 1 — The more represented results for using CAM to visualize the discriminative process of the neural network. (nodules A–D): examples of CNN model incorrectly predicting the benignity and malignancy of nodules. (nodules E–J) examples of CNN model for accurate prediction of nodal benignity and malignancy. As shown in nodule H, the ROI heat values used to determine malignancy is low, but the ROI heat values used to determine non-benign is high, which can be explained by the fact the CNN determines this case as malignant by referring more to the surrounding area than to the nodal region. What is more, we can learn from nodule I that the CT images of this case obviously have texture noise that is not present in other cases, but the CNN still correctly detects the ROI and makes a judgment, which reflects the robustness of the CNN model. CAM, class activation map; CNN, convolutional neural network; ROI, region of interest; CT, computed tomography. [file Image_1.tiff]

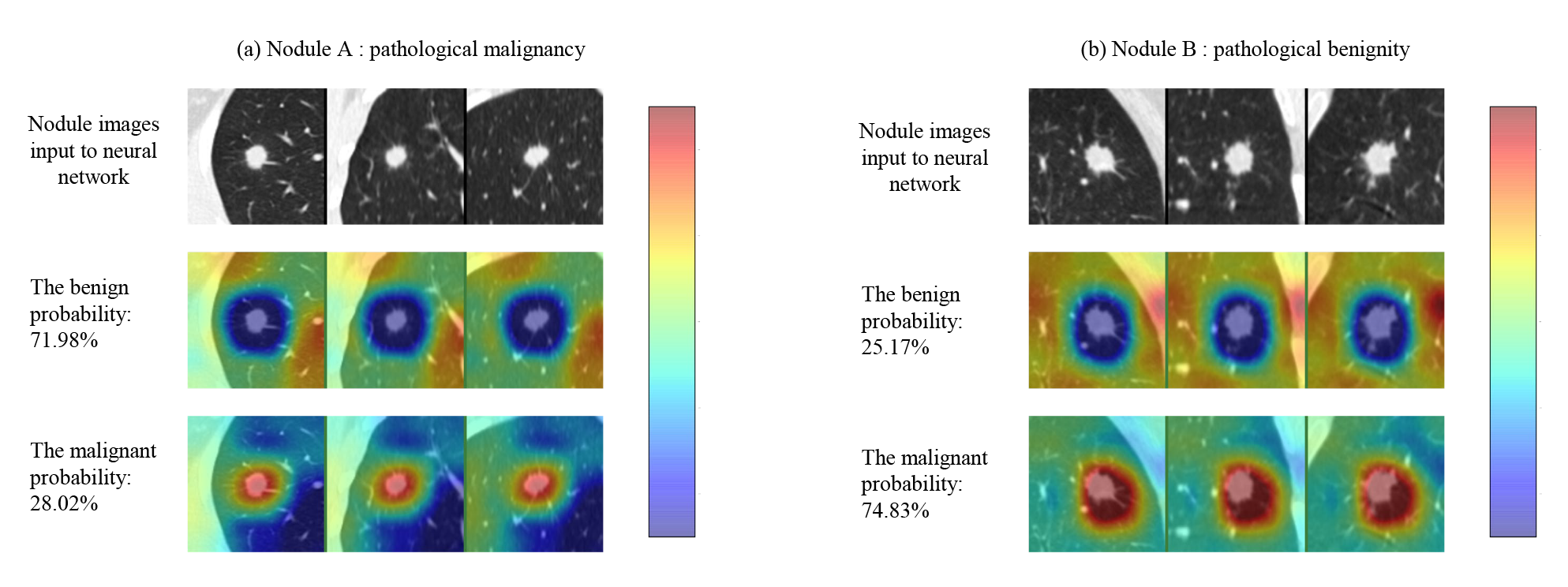

Supplement: Supplementary file 4 [file Image_2.tif]

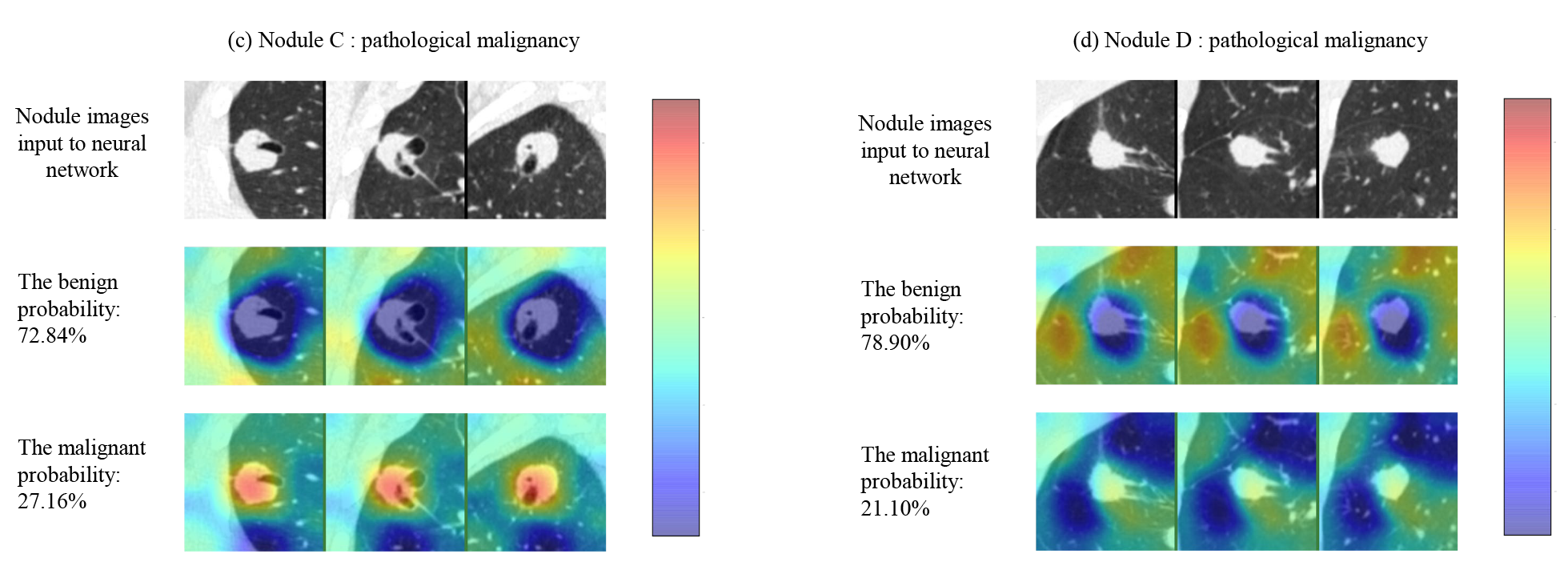

Supplement: Supplementary file 5 [file Image_3.tif]

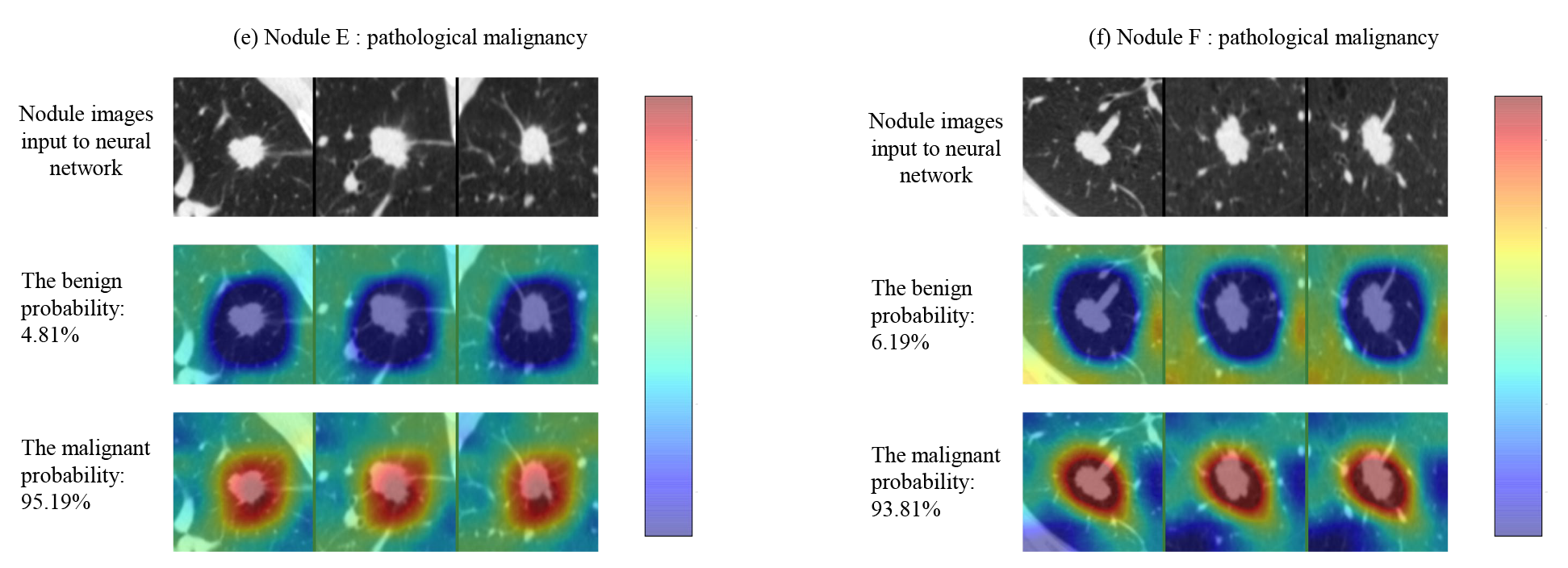

Supplement: Supplementary file 6 [file Image_4.tif]

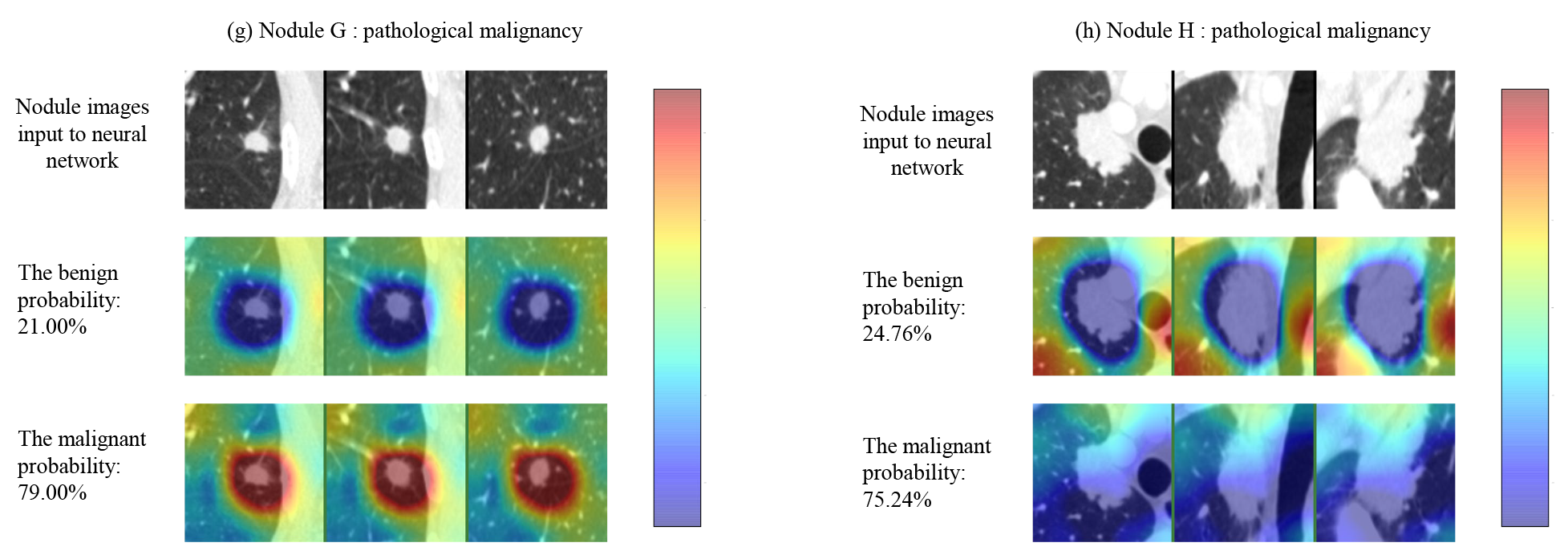

Supplement: Supplementary file 7 [file Image_5.tif]

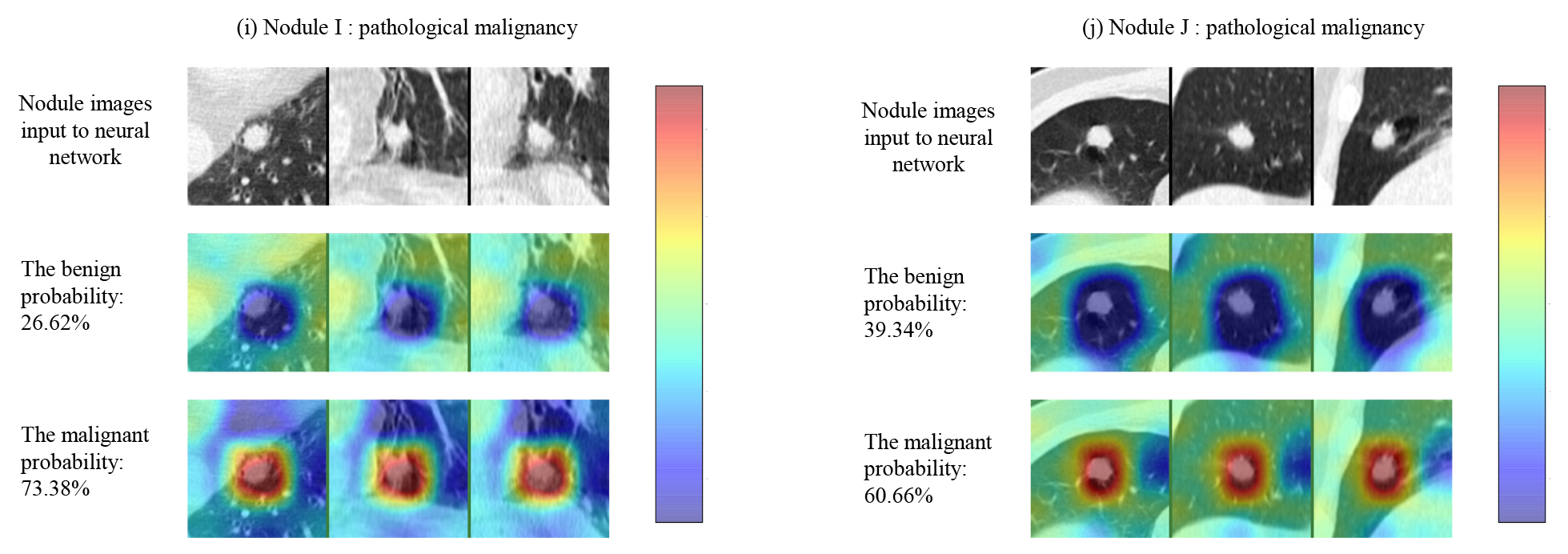

Supplement: Supplementary file 8 [file Image_6.tif]
