# Supplementary material for: Convolutional Neural Network-Based Diagnostic Model for a Solid, Indeterminate Solitary Pulmonary Nodule or Mass on Computed Tomography
Source: Front Oncol. 2021 Dec 21;11:792062. doi: 10.3389/fonc.2021.792062 (PMC8724915; doi:10.3389/fonc.2021.792062)
Supplement: Supplementary file 9 [file Table_1.docx]

Supplement Table 1. CT parameters

| CT scanners | Somatom Definition AS | Brilliance 40 |
| --- | --- | --- |
| Manufacturer | Siemens Medical System | Philips Medical Systems |
| Country | Germany | Netherlands |
| Tube voltage | 120 KV | 120 KV |
| Tube current | 200 mAs | 200 mAs |
| Detector | 64 × 0.625 mm | 40 × 0.625 mm |
| Pitch | 1.0 | 0.4 |
| Matrix | 512 × 512 | 512 × 512 |
| Layer thickness | 1.0 mm | 1.0 mm |
| Increment | 0.7 mm | 0.7 mm |

Supplement Table 2: Confusion matrix of the diagnoses with the CNN model, radiologists alone, and radiologists with the CNN model in each cohort

|  | Final pathological diagnosis(n=459) | | | | | |
| --- | --- | --- | --- | --- | --- | --- |
|  | Training set(n=366) | | Validation set(n=46) | | Test set(n=47) | |
|  | Benign  (n=146) | Malignant  (n=220) | Benign  (n=18) | Malignant  (n=28) | Benign  (n=19) | Malignant  (n=28) |
| **the CNN model** | | | | | | |
| Benign | 122(84) | 24(11) | 14(78) | 4(14) | 15(79) | 4(14) |
| Malignant | 24(16) | 196(89) | 4(22) | 24(86) | 4(21) | 24(86) |
| **Radiologists Alone** | | | | | | |
| Benign | 63(43) | 41(19) | 6(33) | 6(21) | 8(42) | 5(18) |
| Malignant | 83(57) | 179(81) | 12(67) | 22(79) | 11(58) | 23(82) |
| **Radiologists with the assistance of CNN model** | | | | | | |
| Benign | 119(82) | 12(5) | 14(78) | 2(7) | 16(84) | 3(11) |
| Malignant | 27(18) | 208(95) | 4(22) | 26(93) | 3(16) | 25(89) |

NOTE: all values shown as frequency (%)

Abbreviations: CNN, conventional neural network.
